# Demarcation of Prime Farmland Protection Areas around a Metropolis Based on High-Resolution Satellite Imagery

**DOI:** 10.1038/srep37634

**Published:** 2016-12-21

**Authors:** Nan Xia, YaJun Wang, Hao Xu, YueFan Sun, Yi Yuan, Liang Cheng, PengHui Jiang, ManChun Li

**Affiliations:** 1 Jiangsu Provincial Key Laboratory of Geographic Information Science and Technology, Nanjing University, Nanjing, 210093, China; 2 Department of Geographic Information Science, Nanjing University, Nanjing 210093, China; 3 Collaborative Innovation Center for the South Sea Studies, Nanjing University, Nanjing 210093, China; 4 Collaborative Innovation Center of Novel Software Technology and Industrialization, Nanjing University, Nanjing, China

## Abstract

Prime farmland (PF) is defined as high-quality farmland and a prime farmland protection area (PFPA, including related roads, waters and facilities) is a region designated for the special protection of PF. However, rapid urbanization in China has led to a tremendous farmland loss and to the degradation of farmland quality. Based on remote sensing and geographic information system technology, this study developed a semiautomatic procedure for designating PFPAs using high-resolution satellite imagery (HRSI), which involved object-based image analysis, farmland composite evaluation, and spatial analysis. It was found that the HRSIs can provide elaborate land-use information, and the PFPA demarcation showed strong correlation with the farmland area and patch distance. For the benefit of spatial planning and management, different demarcation rules should be applied for suburban and exurban areas around a metropolis. Finally, the overall accuracy of HRSI classification was about 80% for the study area, and high-quality farmlands from evaluation results were selected as PFs. About 95% of the PFs were demarcated within the PFPAs. The results of this study will be useful for PFPA planning and the methods outlined could help in the automatic designation of PFPAs from the perspective of the spatial science.

Farmland is the foundation of human survival and social development and it has expanded rapidly in some regions to meet the needs of rapid population growth and food consumption^1^,^2^,^3^. However, such expansion also brings about ecological deterioration and environmental degradation^4^,^5^. Conversely, urbanization and infrastructure construction contribute to considerable loss of farmland, and unsustainable land management hinders agricultural development^6^,^7^. To resolve this dilemma of increased demand for food production and restricted farmland expansion, the protection of high-quality farmland is an optimal choice^1^,^7^.

China is the most populous and largest developing country in the world. To resolve the prominent contradiction between humans and land, the “Regulations on the Protection of Prime Farmland” (abbr. PF Regulations) was proposed by the State Council in 1994 and they have since been legally recognized as a basic state policy^8^. Prime farmland (PF) is defined as high-quality farmland with high productivity, which has been granted permanent protection according to the “Land Administration Law of the PRC” (abbr. Land Law)^9^. Only key construction projects can expropriate relatively low-quality PF and the farmland requisition–compensation balance must be strictly observed^10^. The PF designation has played a key role in farmland protection for a long time^11^,^12^. Additionally, PF could be considered to have many functions, such as grain production, social security, cultural education, ecological protection, and space obstacles of urban expansion^13^,^14^. Therefore, PF should not be confined to the narrow scope of traditional farmland for purpose of market economy and modern agriculture^8^. In conclusion, PFs should encompass multiple types of land suitable for cropping or that need special protection, such as land for supporting farming facilities, and experimental fields for agricultural research and development^10^.

Generally, PF patches are not directly connected because of access roads for farm machinery, rural residences, and water conservancy facilities (canals or ditches)^15^, which can lead to the damage and physical fragmentation of the farmland^16^,^17^. Thus, it is essential to consider such plots within PF protection, and they should be uniformly managed with PFs by delineating a special protection zone. PF protection areas (PFPAs) are intended to protect both PFs and other related areas (including built-up land, roads and waters)^8^. The designation of PFPAs is the most important task for preserving high-quality farmland^12^,^15^. PFPAs can improve land conservation and strengthen the improvement of land quality by centralized management^10^, and dispersed PF plots can be organized well through land use planning^12^. However, PFPA is not a geographic entity but a virtual space of planning concept, which means the distribution of PFs in reality cannot change just after the demarcation of the PFPAs. Overall, PFs and PFPAs are the products of the policies, aiming at the mandatory protection of high-quality farmlands. Thus the research on the related policies are common, but the studies, considering spatial relationships, are rare.

In China, farmland loss is inevitable as a sacrifice for economic development and food production has declined in recent years^10^. Interestingly, high quality farmland is preferentially occupied by rapid urban expansion in many areas of China^13^,^18^. Farmland quality has diminished dramatically^19^,^20^ and the protection of farmland quality should be given greater attention^15^. Therefore, the researches on designation and protection abilities of PFs and PFPAs are needed urgently. However, few studies have involved the demarcation framework from imageries and its spatial planning. Traditional demarcation methods are based on field measurements or visual interpretation of remote sensing images^21^, but such methods require considerable time and efforts. It has proven effective to integrate remote sensing (supervised classification) and geographic information system (GIS) techniques to zone PFPAs automatically^22^. Automatic interpretation of remote sensing imagery can provide rich geographical information of land use which can facilitate our understanding of its trends^23^,^24^,^25^. Then, the land suitability method could be introduced to select superior farmland as PFs for the following GIS analysis, such as LESA method^26^, IGAS analysis^27^, and FLOWA model^28^. After getting the PF information, GIS spatial analysis can be used to directly plan and design the PFPA^29^,^30^. However, existing researches have mainly focus on the demarcation of PFPA from the medium-resolution imagery in large region^13^,^14^,^22^. Although high-resolution satellite imagery (HRSI) has many advantages^31^,^32^, the relative researches are absent. Additionally, relevant studies on PFPA demarcation by GIS analysis are common, hardly any studies has focused on the landscape connectivity of the PFs and discussed the effects on spatial coefficients in the PFPA demarcation.

Shanghai is a metropolis typical of the new urbanization in China and it has experienced an unprecedented level of the conversion of farmland for urban development^33^,^34^. Although the farming condition in this study area is good, problems related to farmland are also very representative and noteworthy^35^. This study combined remote sensing technique (land use classification), the LESA method (quality evaluation) and GIS analysis (PFPA demarcation). The primary aims were (1) to propose a set of methods to demarcate PFPAs using high-resolution satellite imagery from the perspective of spatial science, (2) to analyze the conditions of PF protection in Shanghai, and (3) to provide a spatial analysis reference for the extraction of PF and the designation of PFPAs.

## Materials and Methods

### Study Area and Research Data

**Figure 1:**
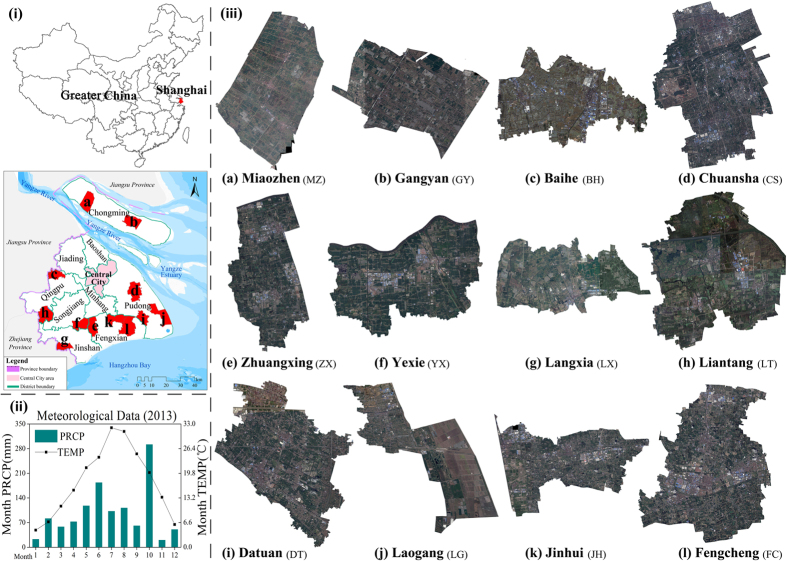
(**i**) Location of Shanghai in Greater China and location of the 12 studied towns in Shanghai, (**ii**) meteorological data of Shanghai for 2013, and (**iii**) remote sensing images of the 12 studied towns. The map data and the satellite images were supported by the Shanghai Institute of Geological Surveys (SIGS). The figure was generated by N.X. and Y.W. using ArcMap 10.0 (http://www.esrichina.com.cn/).

The study area was the municipality of Shanghai (30.67°–31.88°N, 120.87°–122.2°E), which is located in eastern China and has an area of 6340 square kilometers. Shanghai is the economic, financial, and trade center of China, with an average elevation of about 4 m above sea level. The region has a subtropical humid monsoon climate with an annual average temperature of 17.6 °C. The superior natural conditions, developed economic situation, and advanced agricultural technology contribute to the modernized agricultural sector. Shanghai has 16 districts and 1 county, and the farmland is distributed mainly in five of the eight suburban districts (Qingpu, Fengxian, Songjiang, Jinshan, and Pudong New District) and Chongming County. According to the Land Law^9^, the PFPA boundaries should be demarcated by village (county) units. So we chose 12 towns in these 6 districts (county) (Fig. 1) that have well-developed agriculture, especially farming. Table 1 shows the basic situation of each selected town (statistical yearbooks, http://www.stats-sh.gov.cn/). Yexie in Songjiang District has a relatively small agricultural population because of the new family farm pattern called the “Songjiang Mode”.

**Figure 2:**
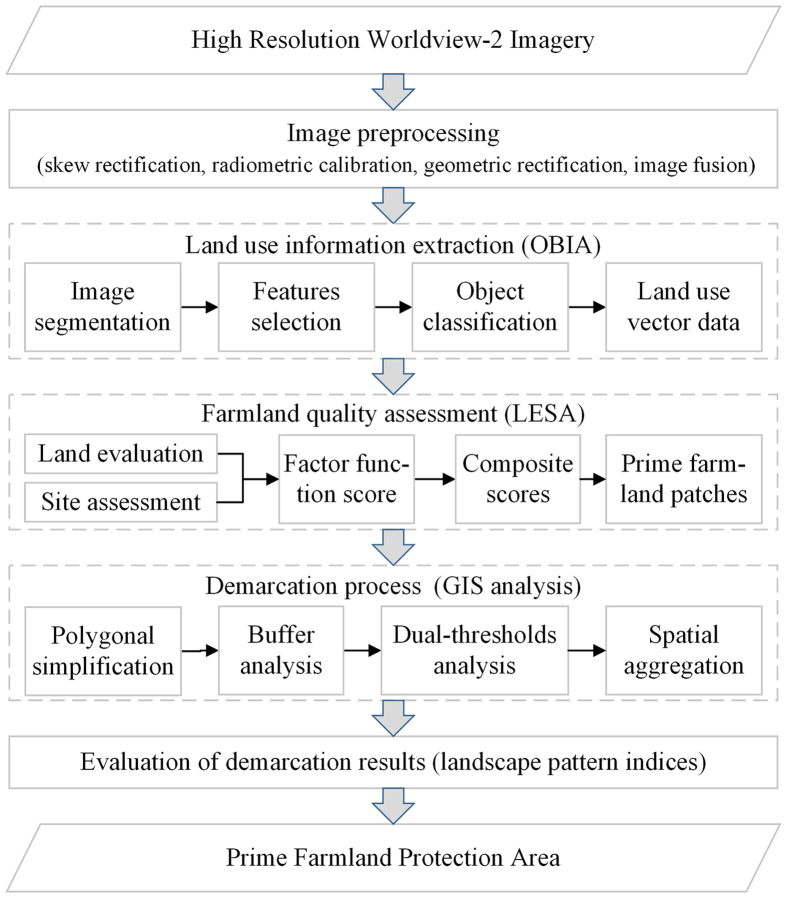
Flow chart of the demarcation of the PFPA from the HRSIs. The figure was generated by N.X. and Y.Y. using Microsoft Visio 2013.

An overview of the PFPA demarcation procedure in this study was displayed in Fig. 2. The commercial high-resolution satellite imageries (HRSIs) was provided by the Shanghai Institute of Geological Surveys (SIGS). The satellite images were acquired by the WorldView-2 (WV2) satellite, which has 2-m spatial resolution and 16.4-km swath width at nadir for eight multispectral bands^36^. The images, acquired from May to September 2013, were selected based on cloud-free conditions over Shanghai. The image preprocessing was conducted by SIGS, mainly including skew rectification of all the imageries to the same view angle, radiometric calibration using FLAASH in ENVI 4.8, geometric rectification using ground GPS points, and image fusion (color balancing). Because of the data confidentiality management in SIGS, the high-resolution WV2 true color composite images (Red/Blue/Green bands vs. R/G/B) could be used to extract farmland. Then, the images for all of the 12 target towns were clipped from the satellite images using administrative division data from 2013.

### Land use information extraction from HRSI

The land use information was extracted from the HRSIs using objected-based classification (OBIA), which could reduce the detriment caused by the heterogeneous mosaics of small features in semi-urban environments^37^ and overcome the problem of low accuracy in conventional pixel-based classification methods^38^. OBIA uses image segmentation to produce discrete image objects that are partly homogeneous and can be used as basic units of classification^39^. When segmentation, OBIA allows the introduction of both spectral and spatial features, which makes both the internal homogeneity of split objects and the heterogeneity of different split objects become optimal^40^. Many studies have shown that OBIA can exploit the advantages of HRSIs fully and improve classification accuracy, e.g., in applications such as tree classification^31^, building extraction^38^, and coastal vegetation extraction^41^. To extract farmland information from the WV2 images, the flowing workflow was implemented: (1) segment images at multiple scales, (2) use the stratified random scheme to obtain reference data and use the Gini index to select appropriate features, (3) classify the segmented images based on the selected features using a random forest (RF) classifier, which is a robust machine-learning classifier^42^, and (4) evaluate the accuracy using an area-based method (Supplementary Approach A). We used the eCognition 8.7 software (http://www.ecognition.com/) to implement the image segmentation and process image classification. When choosing the related parameters, we performed repeated trials and considered previous studies^39^,^43^,^44^. The final classification results of land use patches (vector data) would be evaluated by overall accuracy (including user’s and producer’s accuracy) and kappa coefficient.

### Farmland quality assessment

After getting the land use information, the LESA method was applied to the quantization of farmland quality. The LESA framework was introduced by the USDA Soil Conservation Service^26^, and it focuses on the stability and sustainable production capability of the farmland^45^. The LESA framework contains two components: (1) the land evaluation (LE) component reflects the rating of the natural condition of the farmland; (2) the site assessment (SA) component reflects the socioeconomic suitability of the farmland^15^ and the probability of its transition to non-agricultural use^46^. In this study, LE component was approximately reflected by the natural gradation data of farmland from SIGS, and SA component included the distance to built-up land, density of road networks, density of irrigation water, and distance to the farmers’ market^15^. The natural quality of the farmland and its socioeconomic suitability are considered generally equally important in demarcating PF. The LE and SA component results were calculated separately and their scores were normalized to 0–10, where 10 had the highest quality grade. After obtaining the composite scores for each farmland patch (Supplementary Approach B), a cumulative frequency histogram curve (considering area) was established. Combining the natural breakpoint method and the related stipulation (minimal proportion of farmland area selected as the PF^8^,^9^), different threshold values were obtained for the PF selection of each town. Then the high-quality farmland patches, greater than a certain proportion, were selected as the PF and could be used to demarcate the PFPA.

### Demarcating prime farmland protection areas

**Figure 3:**
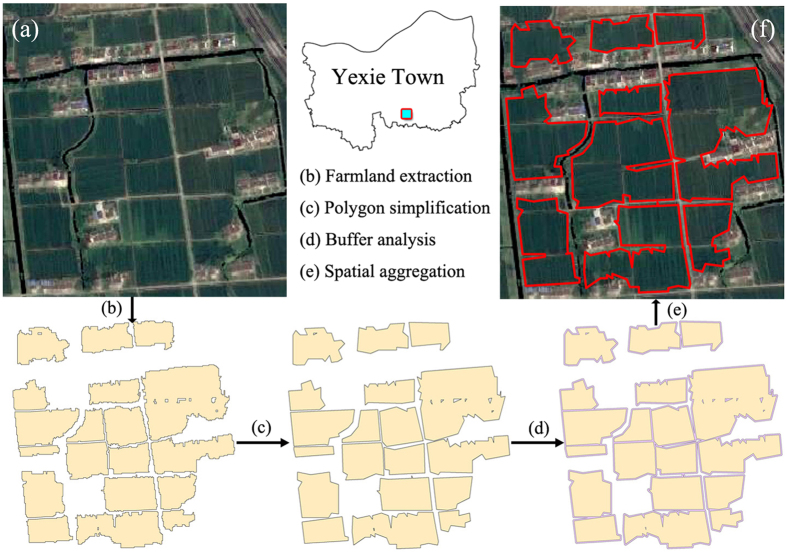
Sketch of the GIS spatial analysis process. The process begins with (**a**) high-resolution satellite images and ends with (**f**) PFPA demarcation result. The figure was generated by N.X. and Y.W. using ArcMap 10.0 (http://www.esrichina.com.cn/) and Adobe Photoshop CS5 (http://www.adobe.com/cn/products/photoshop.html). The map data and the satellite images were supported by the Shanghai Institute of Geological Surveys (SIGS).

Based on PF selection results from the LESA framework, the PFPAs were demarcated using GIS spatial analysis, which was widely used in the ecological planning^47^, natural evolution process^48^, urbanization process^49^, and rural landscape evaluation^50^. Our study included three types of GIS spatial analysis methods (Fig. 3): (1) polygonal simplification to simplify the PF polygons, (2) buffer analysis to analyze the proximity of PF patches, and (3) aggregation analysis to form the PFPA boundaries (Supplementary Approach C). According to the demarcation methods, PF patches within a certain distance (buffer distance threshold: *D*_b_) can be aggregated as PFPAs, and relatively small PFPA patches (minimal area threshold: *A*_m_) were eventually deleted. In order how these two threshold values influence the demarcation results, we gathered the statistics on the proportion of PFs delimited in PFPAs under different threshold combinations (distance: 0–16 m range, 0.2 m step size; area: 0–80 mu range, 1 mu step size; “mu” is the Chinese characteristic area unit, 1 mu ≈ 667 square meters), and established the dual threshold matrices for different towns. Since the distance to urban areas directly determines the amount and distribution of farmland loss^15^, the 12 towns will be grouped into suburban (CS, DT, LG, JH, and FC) and exurban area (MZ, GY, BH, LT, ZX, YX, and LX) according to their spatial distribution in Shanghai and built-up land area. This group mode has also been validated by the correlations of their dual threshold matrices (Supplementary Approach D1). Comprehensively considering the actual situation, landscape connectivity, and the related policies (about 95% PFs had to be demarcated in the PFPA), we gave an optimal suggestion of the threshold settings for the study area after some trials (Supplementary Approach D2).

Although the demarcated PFPAs were expected to be less fragmented than the original PFs in distribution (elimination of small discrete patches and merge of nearby patches), some non-farmlands were still included within the PFPAs. To quantitatively evaluate the demarcation results of PFPAs, we used landscape pattern indices to compare their spatial pattern though the PFPAs do not exist in the real space (Supplementary Approach E). Landscape pattern indices can illustrate the relationship between landscape patterns and ecological processes at landscape level^51^, and advance the understanding of the ecological functioning of landscapes^52^. According to the related researches^16^,^49^,^53^ and repeated experiments, four most reasonable and representative indices (COHESION, MESH, LSI, and PAFRAC) were selected to prove that expectation in spatial patterns.

## Results

### Land use information from HRSI

The scale parameter for the image segmentation was selected as 80 in our study area. The weight of color/shape was set to 0.9/0.1 because the spectral factor was considered the major segmentation factor. The weight of smoothness/compactness was set to 0.5/0.5 and the weights of the R/G/B band layer were set equally to 1/3. According to the variable importance of different features in the classification (Supplementary Table 1), all the spectral features, and only five or six types of textural and shape features were selected^43^. The reference data for classification were manually photointerpreted from the WV-2 imageries in this study and from the imageries available within the Google Earth. The typical land use types for the classification were displayed in Supplementary Table 2. According to the statistics of the reference data, the spectral separability for different pairs of land use types were basically >1.8 (Jeffries-Matusita distance), except some pairs including farmland-garden land, farmland-forest, and farmland-road (about 1.7). That meant appropriateness and strong spectral separability of the reference data for the classification. The imageries in this study were classified into seven land use types: farmland, built-up land, water body, road, forest land, garden land, and other land (Supplementary Fig. 1, Supplementary Table 3).

The confusion matrix for the land use classification in MZ town was displayed as an example in the Supplementary Table 4. As shown in Table 2, the overall accuracy (OA) of the classification results for each town is approximately 80% and the kappa coefficient (KC) is about 0.7. The user’s accuracy and the producer’s accuracy of the farmland class were all >80%, which indicate that farmland extraction results are robust. According to the classification results, the proportion of farmland area ranges from 23.68% to 56.48% (Table 2). And the farmland area in each town shows positive correlation with the gross output value of agriculture and agricultural population (Table 1).

### Farmland quality in Shanghai

After obtaining the farmland patches from the WV2 images (2013) for the 12 towns, we used the LESA framework to extract PF patches. According to the evaluation results (Supplementary Fig. 2-1, 2-2, 2-3), the natural quality (LE) of ZX, YX, and LT scores relatively low and the other nine towns score similar values. The socioeconomic condition (SA component) of MZ, GY, and LT score the highest, and ZX and CS score relatively low. We used the area-weighted average LESA scores (AWSs), with full marks of 10, to evaluate the farmland quality of the 12 towns. The AWSs of the towns range from 5.68 to 8.91, and two towns in Chongming County score the highest. According to the scores of the different towns, we selected the farmland with the highest scores to be potential PFs (Supplementary Fig. 3). The evaluation criterion for the 12 towns was the same but slightly different evaluation score thresholds were applied to different towns. According to the Land Law, provinces or municipalities should account for >80% of the farmland within their PF administrative areas. According to our results, the proportion of farmland selected as PF (pF) ranges from 71.13% to 89.76% (Table 2).

### Demarcation of the PFPAs

**Figure 4:**
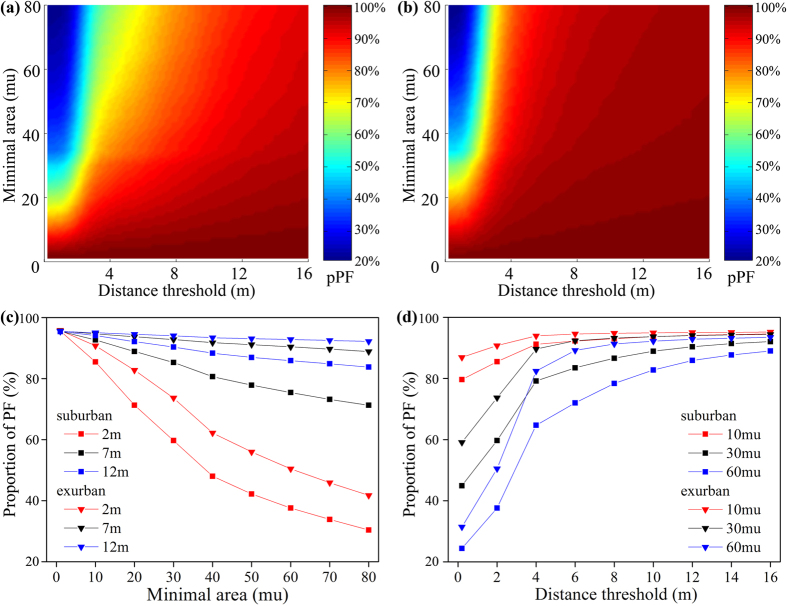
Proportion of PFs demarcated in PFPAs (pPF, z-axis) in terms of the relationship between the buffer distance (x-axis, m) and minimal area threshold (y-axis, mu, 1 mu ≈ 667 square meters). Composite results of (**a**) suburban areas and (**b**) exurban areas, and pPF (**c**) under certain buffer distances and (**d**) under certain minimal areas. The figure was generated by H.X. and L.C. using Matlab R2014a (http://cn.mathworks.com/).

**Figure 5:**
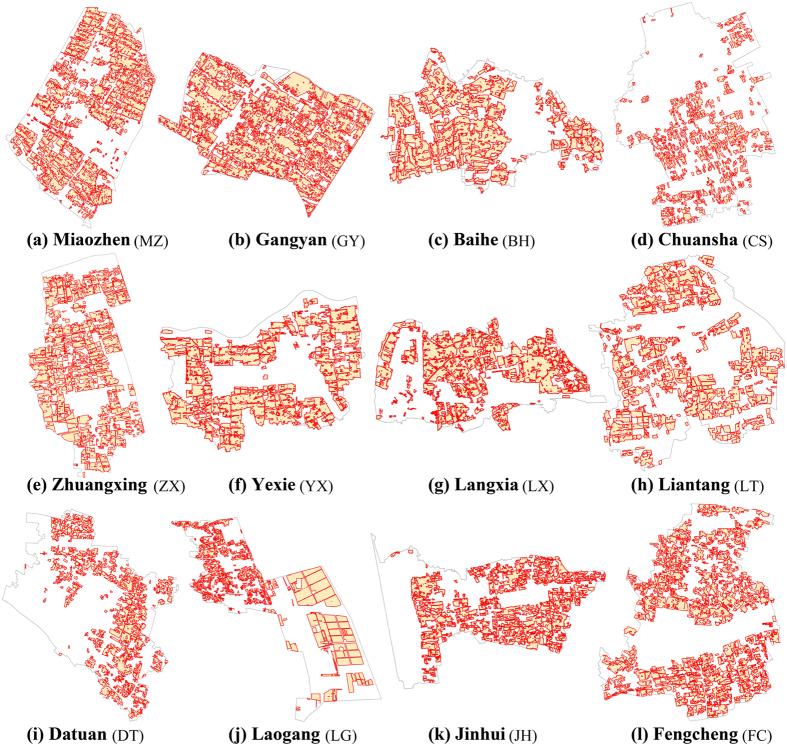
PFPA demarcation results for the 12 studied towns in Shanghai. Threshold values and statistical results for each town can be found in Table 3. The figure was generated by H.X. and L.C. using ArcMap 10.0 (http://www.esrichina.com.cn/). The map data was supported by the Shanghai Institute of Geological Surveys (SIGS).

We calculated the mean value of the dual-threshold matrices of the two groups (urban area and exurban area) separately (Fig. 4a,b). The figure clearly show the relation between two thresholds and the pPF (proportion of PFs demarcated in PFPAs): the distance threshold shows positive correlation with pPF; however, the minimal area threshold shows negative correlation. The value of pPF is greater in the exurban areas than in the suburban areas under the same threshold combinations (Fig. 4c,d). The threshold values of the12 towns are shown in Table 3. The distance threshold and minimal area thresholds are approximately 9 m and 20 mu (about 1.33 × 10^4^ square meters) for the suburban areas respectively, and 7 m and 35 mu (about 2.33 × 10^4^ square meters) for the exurban area respectively. From the practical implications, these thresholds indicate that: (1) the average rural road width is approximately 7 m in the exurban area and 9 m in the suburban area; (2) the average farmland size in suburban area is smaller than that in exurban area. These differences are obvious for a single city and can be understood easily according to different social economic development level of suburban and exurban area. However, there are still slight deviations of threshold selections within the suburban group and exurban group (Table 3). The demarcation results based on different threshold settings for different towns are shown in Fig. 5. The total area of the PFPAs ranges from 11.83 to 40.71 square kilometers and GY, MZ, and FC have the biggest PFPA area (Table 3). The average area of the PFPAs ranges from 0.24 to 0.44 square kilometers and MZ, GY, and YX have the biggest average PFPA area. The proportion of PFs delimited in PFPAs (pPF in PFPA) ranges from 94.53% to 96.32% according to actual situations in different towns, which indicates that the PFPAs include almost all the PFs and satisfy the related policy.

The quantitative evaluation of the demarcation of the PFPAs using landscape pattern indices is shown in Supplementary Table 5. The COHESION and MESH show an obviously high landscape connectivity for the demarcated PFPAs in all 12 towns. The LSI shows wide numeric decrement, indicating a regular landscape shape for PFPAs. The PAFRAC index shows a small value, indicating the shape simplicity and elimination of small perforations inside the PFPAs. The regular shape and strong connectivity of the PFPAs are convenient for conducting centralized management.

## Discussion

### Existing problems in this study

HRSIs can advance studies on land use distribution and object information extraction^31^. The addition of the texture feature to the classification of HRSIs can overcome the lack of spectral resolution and supply supplementary information about the properties of the images^38^; however, some problems of classification remain. The low variable importance of the shape and texture features may be related to correlations with spectral features or intercorrelations between each other. That deserved a thorough study in the future. Farmland is sometimes difficult to distinguish from forest and garden land because of their similar spectral characteristics. Due to the data confidentiality management in SIGS, the WV-2 imagery in our study lacks spectral information in VNIR, which can potentially improve the classification accuracy using vegetation index^32^. This problem could also be solved by data fusion of high-resolution imagery and medium-resolution imagery with high spectral information (Landsat-8)^54^. Furthermore, shadowed areas are occasionally seen in urban areas and on the fringes of forest land. The widespread distribution of agricultural facilities in Shanghai can also confuse the classification results. In order to resolve the above problems, we performed some manual interpretation to fix area boundaries that had apparent errors and to adjust plot properties.

The gradation of farmland is a standard for measuring farmland quality with Chinese characteristics. Because of the lack of land environmental data, we used the natural gradation of farmland data to approximate the LE component. Apart from the six factors in the traditional LE component, the gradation data include external environmental conditions. The gradation data are based on separate farmland plots with divisions of 1–15 that can only reflect the relative quality by sacrificing absolute accuracy. The SA component in our study lacked some socioeconomic suitability data, such as farmland yield, planting input (agricultural socioeconomic data, such as agricultural water conservancy, machinery input and means of production), and farmers’ market trading data. Actually, these factors has little spatial difference in Shanghai and only slightly influenced the farmland quality assessment results in the study area.

The demarcation process using GIS spatial analysis seems too complicated. The polygonal simplification would lose some accuracy for classification, and we did not evaluated this loss and its effects. The buffer analysis and aggregation consider only spatial distribution of PFs. But actually, the PFPA demarcation is a comprehensive process involving personal interests, local government will, and national land use policy and laws. But these factors are hard to conduct quantitative analysis. For example, although land use policy has significant effect on the quantity and distribution of farmland^55^, the comprehensive policies in text are sometimes hard to be transformed into spatial planning for PFPAs. The dual threshold matrices in this study is hard to understand and not completely scientific. It is not a direct and efficient way to identify the optimal thresholds. These would be our further research interest.

### Significance and Practical applications

The increase of population density and GDP has driven the need for more land in Shanghai^34^, and the PFPAs could be a type of reserve land resource for a long-term perspective. The demarcation of PFPAs, which is central to the management of superior farmland resources, can minimize farmland loss^11^, reduce the fragmentation of management, and improve farmland yields^12^. Although PFPAs would exclude some small PF patches, the demarcation can also improve farmland quality to great extent, improve the economic and intensive use of land, and strengthen the control of land use^10^. The intensity of farmland resources reduces inefficient cultivation and farmland abandonment^56^. Furthermore, demarcation can also help governments implement responsible PF protection and complete basic PF protection indices. For farmers, PFPAs can stabilize their thoughts regarding contracted farmland and increase production^55^. The demarcation of PFPAs is the basis of the PF protection red line (PFRL). The PFRL is the one of the “three lines” in the overall planning of land utilization; the other two are the ecological protection red line and urban development boundaries. The PFRL can effectively implement strict space management and constrain urban expansion^12^. Because of the stability of PFPAs, land transformation models and simulation models can be more credible^57^, and the PFPAs around big cities can control urban sprawl. Moreover, farmland resources in metropolises, especially in China, are often scarce, and PFPAs in such areas can play an ecological function and preserve ecological connectivity^16^. Large farmland areas can also protect biodiversity^58^.

The previous demarcation of the PFPA in Shanghai was manual delineation by on-the-spot mapping and digitalization. The PFPA demarcation was basically in accordance with the national uniform rules^8^. This manual method cannot consider the spatial scientificity (landscape connectivity) and waste much time. Our semiautomatic methods could be useful in demarcating the PFPA in other areas of China. For example, the Yangtze River Delta Economic Zone (including Shanghai) has similar natural and socioeconomic conditions. The successful experience in Shanghai could be easily applied to the practice of demarcations in these area, and related coefficients could be also appropriate. Additionally, our methods and research results have been checked and accepted as a subtopic of research program (“Compilation Technique and Method for the Overall Plan of Land Utilization”) for the *Overall Urban Planning of Shanghai (2015–2040)*. But the PFPA demarcation in this study was not comprehensive for the lack of stakeholders input, such as biological, environmental economic and urban development data. For example, the InVEST model can be useful in habitat quality assessment^59^, and spatial planning^60^,^61^. The future study would take advantage of these models in human development and conservation, and delineate the PFPA more comprehensively and scientifically.

### Application Suggestions

PFs are a precious resource and this article proposes a framework (from the spatial-science perspective) for the demarcation of PFPAs directly from the remote sensing data. The HRSI, better fused with hyperspectral data, can tremendously reduce the effort of *in-situ* measures^25^. When we select PFs from the automatically interpreted farmland information, its natural quality and its location quality should be both considered. Diverse factor combinations for the LESA method should be selected based on the actual situation, just as slope factor needed no consideration in Shanghai for the alluvial plain topography. When demarcating PFPAs, we should consider both the connectivity and the minimal area of PF patches because PFPA boundaries should not segment other surrounding plots and cadastral parcels. As shown in spatial aggregation of PFPAs in our study, different distance and area thresholds should be applied to suburban and exurban regions. In that analogy, PFPA demarcation in different cities could be applied to different distance and area thresholds, as different cities have various urban morphology and development mode. Especially, farmland protection is intended to increase the agricultural population and promote farmers’ incomes^56^. Government should encourage the family farm pattern in order to increase agricultural production, as in Songjiang District where farmland has high quality and connectivity (Supplementary Figs 2 and 4), because this mode can avoid the contradiction of ownership fragmentation and landscape fragmentation^62^.

## Conclusions

This study proposed a method for demarcating PFPAs in an area of rapid urbanization in China using HRSIs. We extracted land use information from the HSRIs using OBIA, and high-quality farmland was evaluated using the LESA framework. Finally, PFPAs were demarcated using GIS spatial analysis. The study area encompassed 12 towns in Shanghai. The HRSIs were first segmented based on spectral and textural features, and then classified into seven types using an RF classifier. The OA of the classification was about 80%, and the KC was 0.7. Then, the farmland quality was evaluated using the LESA framework, which considers both natural and socioeconomic conditions. The towns on Chongming Island, away from urbanized areas, scored the highest. Those farmland patches with high scores were selected as the PF. In this study, 71.13–89.76% of farmland in the various towns was set as potential PFs. Subsequently, we applied GIS spatial analysis to the demarcation of PFPAs, including line simplification, buffer analysis, and spatial aggregation. We gathered the statistical results of pPF under different combinations of distance and area thresholds and classified the 12 towns into suburban and exurban groups. The distance and area thresholds for suburban areas were 9 m and 20 mu (about 1.33 × 10^4^ square meters), respectively, whereas for exurban areas, they were 7 m and 35 mu (about 2.33 × 10^4^ square meters), respectively. The discrepancy between the two areas was mainly because of the unevenness of the farmland distribution and regional development in Shanghai. Finally, 94.53–96.32% of PF was demarcated within the PFPAs. The PFPA demarcation results were evaluated using landscape pattern indices that indicated strong connectivity of the PFPAs.

Further study should focus on the driving mechanisms of the different threshold values in different regions, which would allow the demarcation method to be applicable to other metropolises in China. The policy, laws and urban planning would be considered in the demarcation of PFPA to improve accuracy and reliability. In addition, the accuracy of image classification, objectivity of land evaluation, and scientificity of threshold selection should all be improved. Ultimately, a universal method for the demarcation of PFPAs that considers both science and policy is desirable.

## Additional Information

**How to cite this article**: Xia, N. *et al*. Demarcation of Prime Farmland Protection Areas around a Metropolis Based on High-Resolution Satellite Imagery. *Sci. Rep.* **6**, 37634; doi: 10.1038/srep37634 (2016).

**Publisher's note:** Springer Nature remains neutral with regard to jurisdictional claims in published maps and institutional affiliations.

## Supplementary Material

Supplementary Information

## Figures and Tables

**Table 1:** The 6 districts/county and 12 towns under their jurisdiction.

District/County	Town	Abbr.	Area (km^2^)	GOVA (10^4^yuan)	AP	CSA (km^2^)	CP (10^3^ton)
Chongming	Miaozhen	MZ	95.51	54083	14538	73.02	93.58
	Gangyan	GY	75.11	46448	14622	67.83	103.15
Qingpu	Baihe	BH	57.63	47583	8661	75.31	151.36
	Liantang	LT	93.66	44192	6398	72.15	149.93
Pudong	Chuansha	CS	96.72	50745	9673	29.04	80.55
	Datuan	DT	50.64	44332	8451	24.02	51.09
	Laogang	LG	94.21	49300	9398	54.68	63.54
Fengxian	Jinhui	JH	72.83	45198	9467	57.60	93.69
	Fengcheng	FC	110.65	98032	13382	77.24	120.48
	Zhuangxing	ZX	70.06	56780	5418	68.30	113.59
Songjiang	Yexie	YX	72.54	**42201**	**1316**	40.08	67.43
Jinshan	Langxia	LX	47.87	29982	3957	45.71	81.89

The table introduces the basic situation of every town, including Area, Gross Output Value of Agriculture (GOVA), Agricultural Population (AP), Crop Sown Area (CSA), and Crop Production (CP).

^1^Agriculture consists of planting, forestry, animal husbandry, fishery, and related avocation.

^2^Crop includes food crop, industrial crop and other crop.

^3^Abbr. km^2^: square kilometers.

^4^The AP consists of local registered population and migrant workers.

**Table 2:** The classification evaluation of satellite images and farmland quality assessment results.

Town	Classification results						Assessment results		
	OA(%)	KC	UA_f_ (%)	PA_f_ (%)	FA(km^2^)	pFA(%)	AWS	PFA(km^2^)	pF(%)
MZ	82.84	0.75	90.84	89.61	43.26	45.29	8.78	35.77	82.69
GY	82.21	0.74	89.40	87.44	42.42	56.48	8.91	35.17	82.91
BH	79.06	0.71	86.23	85.43	29.58	51.33	8.16	24.05	81.30
LT	81.97	0.74	88.06	89.04	33.80	36.09	7.16	30.18	89.27
CS	76.39	0.68	80.30	82.65	22.90	23.68	6.08	16.29	71.13
DT	78.25	0.70	85.80	87.58	15.14	29.90	7.43	11.17	73.77
LG	84.36	0.76	89.12	91.39	37.36	39.66	7.99	28.97	77.54
JH	80.26	0.72	86.18	85.84	26.78	36.78	6.39	20.67	77.19
FC	77.38	0.69	82.36	81.87	40.36	36.48	7.28	35.03	86.78
ZX	78.67	0.69	83.41	86.31	34.03	48.57	5.68	28.98	85.17
YX	81.34	0.73	87.64	88.84	32.95	45.42	5.96	29.57	89.76
LX	81.10	0.72	88.01	86.37	23.47	49.03	7.33	18.00	76.69

(1) The indexes of classification results contain ①overall accuracy (OA), and kappa coefficient (KC) of all classification results; ②user’s accuracy (UA_f_) and producer’s accuracy (PA_f_) of the farmland class; ③farmland area (FA, 3. abbr. km^2^: square kilometers) and proportion of farmland area (pFA). (2) The indexes of farmland quality assessment results contains area-weighted average LESA score (AWS, prime farmland area (PFA) and proportion of farmland selected as PF (pF).

**Table 3:** The thresholds value and statistical results for PFPA of each town (the towns’ names are abbreviations and can be referred to Table 1).

Town	Thresholds		removal PF (km^2^)	pPF (%)	QTY of PFPAs	Total Area (km^2^)	Average area (km^2^)
	D_b_(m)	A_m_(mu)					
MZ	6.6	38	1.76	95.08	87	38.03	0.44
GY	6.8	37	1.29	96.32	96	40.71	0.42
BH	6.9	34	1.17	95.14	70	25.44	0.36
LT	6.7	36	1.65	94.53	92	30.78	0.33
CS	9.6	17	0.81	95.02	68	17.41	0.26
DT	9.4	18	0.54	95.21	46	11.83	0.26
LG	9.2	19	1.21	95.84	103	31.06	0.30
JH	8.8	22	1.10	94.67	92	21.72	0.24
FC	9.0	20	1.46	95.83	126	36.40	0.29
ZX	6.8	34	1.42	95.10	83	30.36	0.37
YX	7.2	32	1.37	95.36	77	31.81	0.41
LX	7.0	35	0.93	94.86	49	19.18	0.39

The threshold value consist of buffer distance (D_b_) and minimal area of PFPA (A_m_, 1 mu ≈ 667 square meters). The statistical results of demarcated PFPA consist of the quantity of PFPA patches (QTY of PFPAs), total area, average area of PFPA, the PF area outside the PFPA (removal PF, abbr. km^2^: square kilometers), and proportion of the PF inside the PFPA (pPF).
