# Supplementary material for: Demarcation of Prime Farmland Protection Areas around a Metropolis Based on High-Resolution Satellite Imagery
Source: Sci Rep. 2016 Dec 21;6:37634. doi: 10.1038/srep37634 (PMC5175287; doi:10.1038/srep37634)
Supplement: Supplementary Information [file srep37634-s1.doc]

**Demarcation of Prime Farmland Protection Areas around a Metropolis Based on High-Resolution Satellite Imagery**

*Authors:* Nan Xia^1, 3^, YaJun Wang ^1, 3^, Hao Xu^1, 3^, YueFan Sun ^1, 3^, Yi Yuan ^1, 3^ , Liang Cheng^1, 2, 3, 4*^, PengHui Jiang^1, 3^, ManChun Li^1, 2, 3, 4^**

^1^ Jiangsu Provincial Key Laboratory of Geographic Information Science and Technology, Nanjing University, Nanjing, 210093, China

^2^ Collaborative Innovation Center for the South Sea Studies, Nanjing University, Nanjing 210093, China

^3^ Department of Geographic Information Science, Nanjing University, Nanjing 210093, China

^4^ Collaborative Innovation Center of Novel Software Technology and Industrialization, Nanjing University, Nanjing, China

* Corresponding Author:

Liang Cheng

Professor

Department of Geographic Information Science, Nanjing University

Phone/Fax: +86-25-83597359

Email: lcheng@nju.edu.cn

**Supplementary Figure**


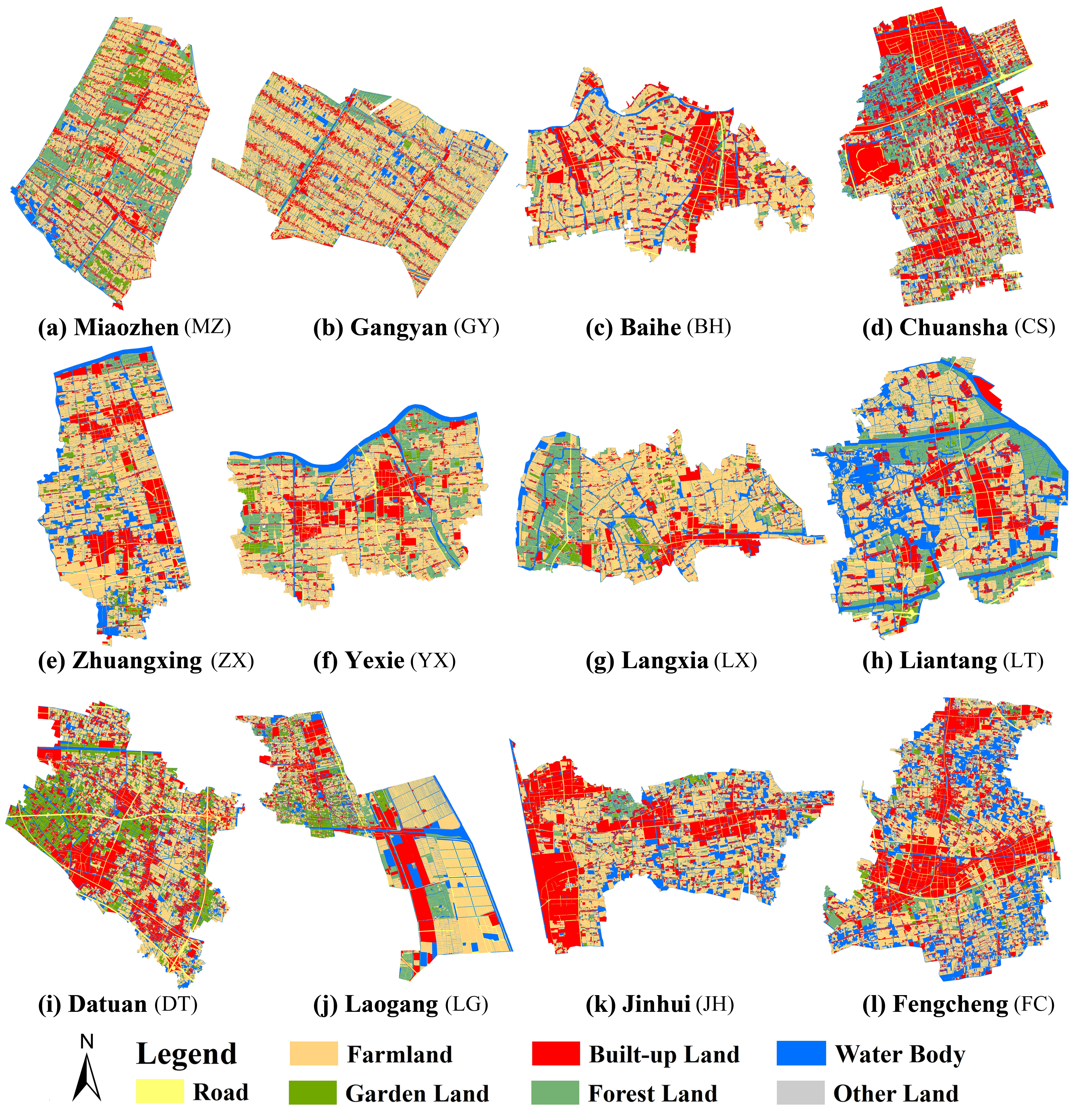


**Supplementary Figure 1.** Classification results of 12 study areas. The land use types include farmland, built-up land, water body, road, garden land, forest land, and other land. The figure was generated by H.X. and Y.S. using eCognition 8.7 (<http://www.ecognition.com/> ) and ArcMap 10.0 (<http://www.esrichina.com.cn/>). The administrative boundaries data were supported by the SIGS.


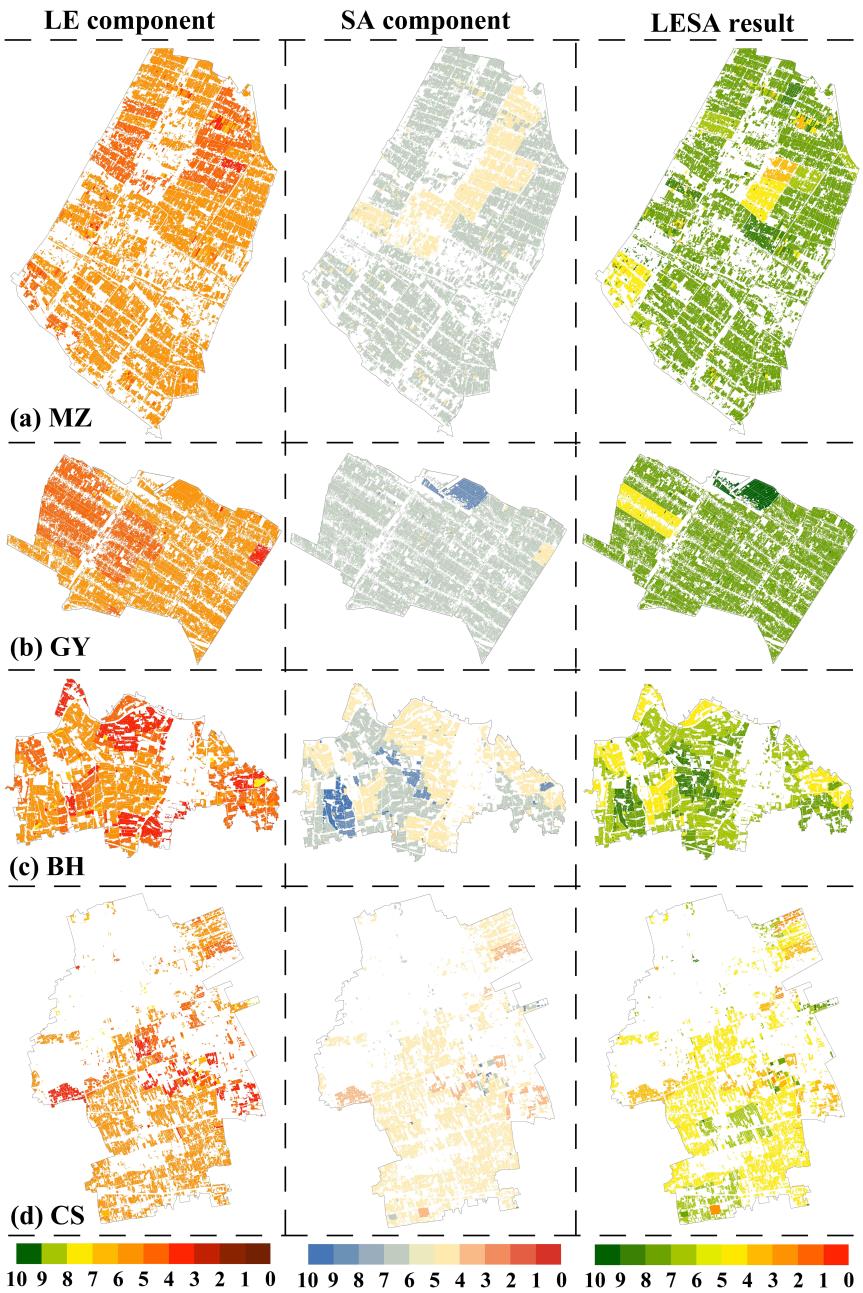


**Supplementary Figure 2-1.** Land evaluation results, site assessment results and LESA results of 4 towns: (a) Miaozhen(MZ), (b) Gangyan(GY), (c) Baihe(BH) and (d) Chuansha(CS). The score 10 has the highest quality grade, and each color represents scores between two integers. The figure was generated by Y.Y. and Y.S. using ArcMap 10.0 (<http://www.esrichina.com.cn/>). The administrative boundaries data were supported by the SIGS.


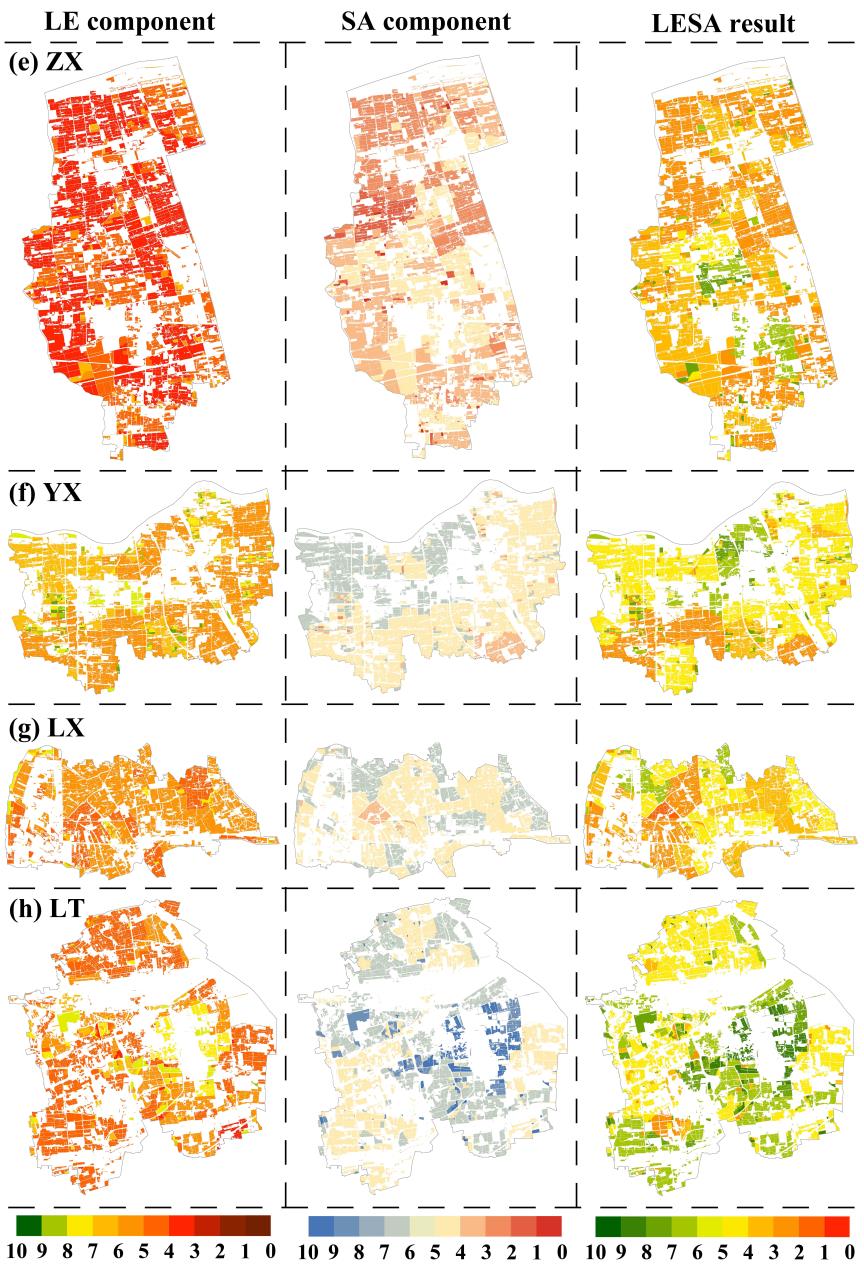


**Supplementary Figure 2-2.** Land evaluation results, site assessment results and LESA results of 4 towns: (e) Zhuangxing(ZX), (f) Yexie(YX), (g) Langxia(LX) and (h) Liantang(LT). The score 10 has the highest quality grade, and each color represents scores between two integers. The figure was generated by Y.Y. and Y.S. using ArcMap 10.0 (<http://www.esrichina.com.cn/>). The administrative boundaries data were supported by the SIGS.


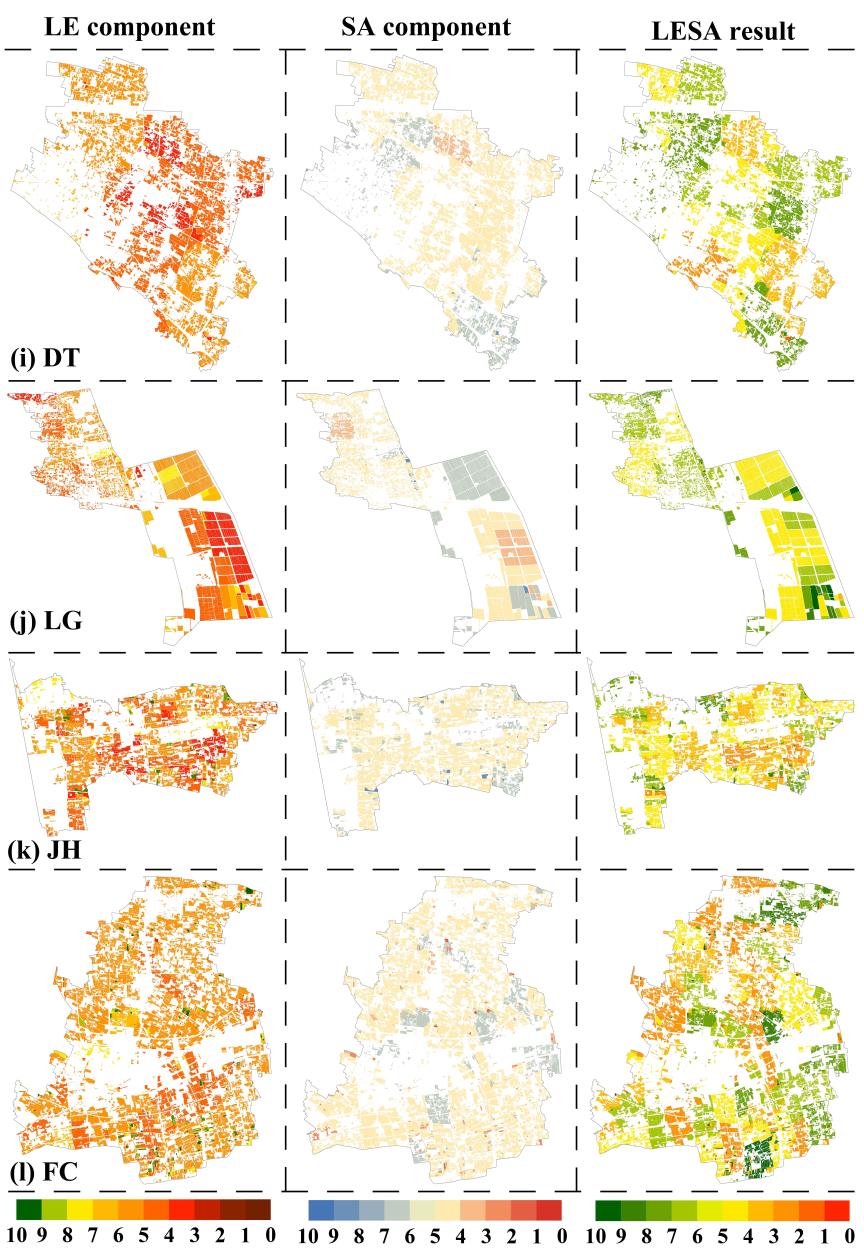


**Supplementary Figure 2-3.** Land evaluation results, site assessment results and LESA results of 4 towns: (i) Datuan(DT), (j) Laogang(LG), (k) Jinhui(JH) and (l) Fengcheng(FC). The score 10 has the highest quality grade, and each color represents scores between two integers. The figure was generated by Y.Y. and Y.S. using ArcMap 10.0 (<http://www.esrichina.com.cn/>). The administrative boundaries data were supported by the SIGS.


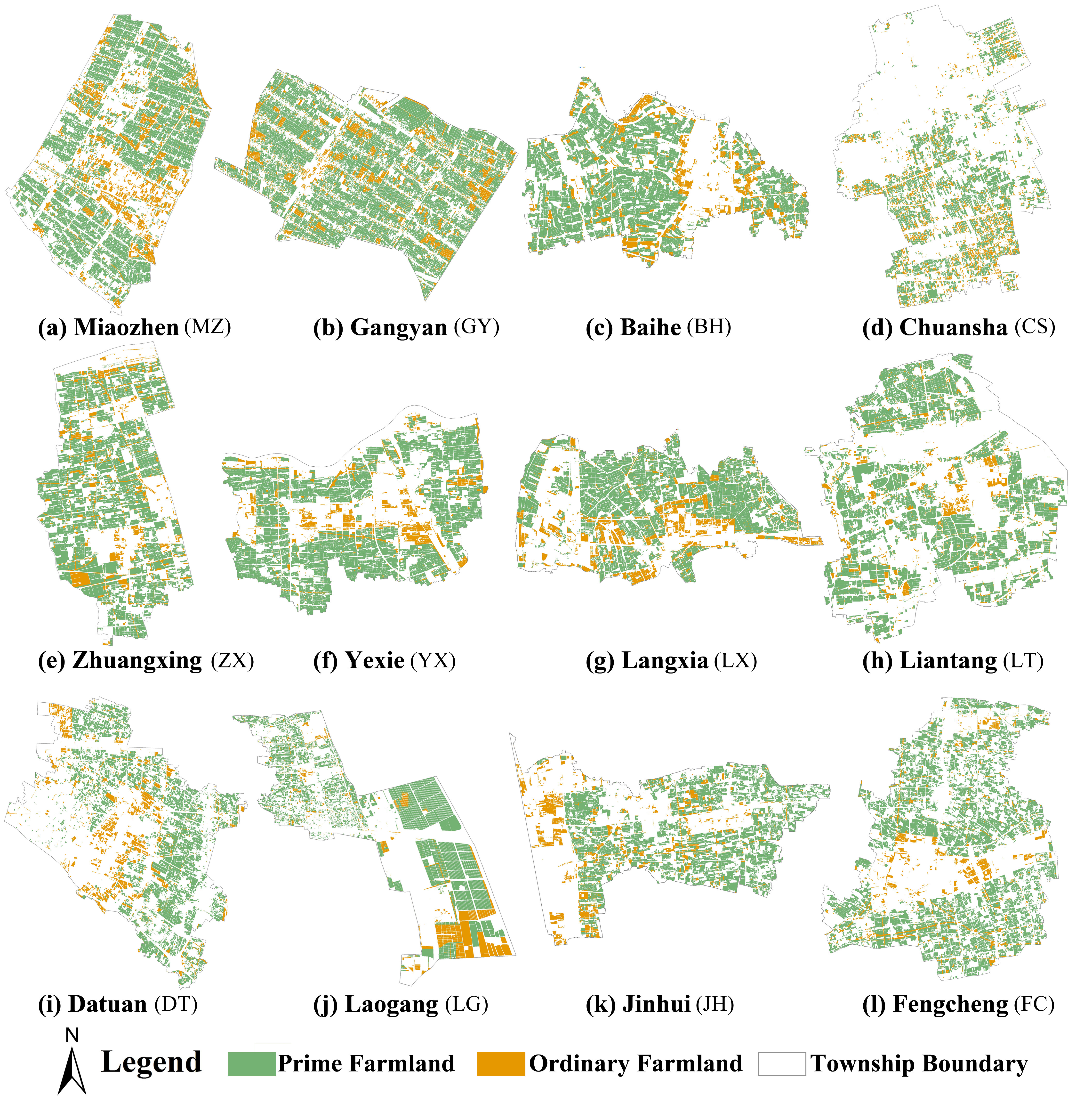


**Supplementary Figure 3.** Prime farmland demarcation results of 12 study areas. The figure shows the relationship of prime farmland (with high scores) and ordinary farmland (with low scores and not suitable for the constructions of prime farmland). The figure was generated by H.X. and Y.W. using ArcMap 10.0 (<http://www.esrichina.com.cn/>). The administrative boundaries data were supported by the SIGS.


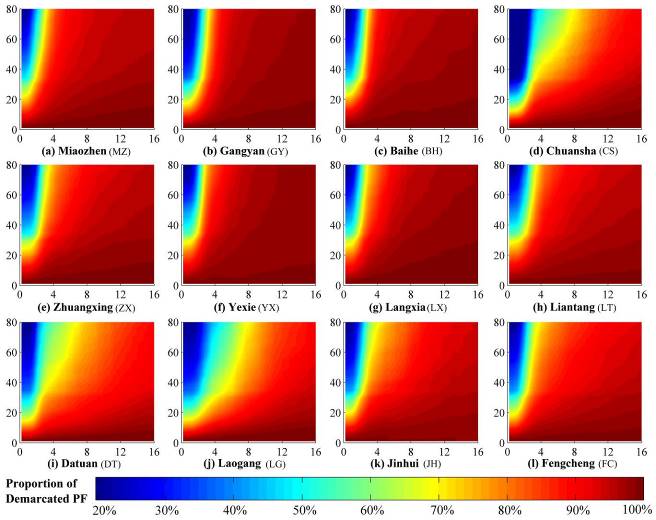


**Supplementary Figure 4.** Proportion of PF demarcated in the PFPA in terms of the relationship between the buffer distance and area threshold. The x-axis shows the buffer distance (unit: meter) and the y-axis shows the area threshold (unit: mu, 1 mu ≈ 667 square meters). The z-axis was shown by hierarchical color. The figure was generated by H.X. and L.C. using Matlab R2014a (<http://cn.mathworks.com/> ).

**Supplementary Table**

**Supplementary Table 1.** The relatively important variables (features) in the RF classification process for 12 towns at the 80 segmentation scale. The symbol √ means higher variable importance. Some features (such as roundness, main direction, asymmetry, elliptic fit, GLCM std. dev., GLCM dissimilarity, GLCM std. dev.) are not displayed for they are relatively unimportant variables for any of the 12 towns.

|  | | MZ | GY | BH | LT | CS | DT | LG | JH | FC | ZX | YX | LX |
| --- | --- | --- | --- | --- | --- | --- | --- | --- | --- | --- | --- | --- | --- |
| S  P  E  C  T  R  A  L | R/G/B  means | √ | √ | √ | √ | √ | √ | √ | √ | √ | √ | √ | √ |
|  | R/G/B  Std. dev. | √ | √ | √ | √ | √ | √ | √ | √ | √ | √ | √ | √ |
|  | max difference | √ | √ | √ | √ | √ | √ | √ | √ | √ | √ | √ | √ |
|  | brightness | √ | √ | √ | √ | √ | √ | √ | √ | √ | √ | √ | √ |
| S  H  A  P  E | area |  | √ |  |  | √ | √ |  | √ | √ |  |  |  |
|  | compactness | √ |  | √ | √ | √ | √ | √ | √ | √ | √ | √ |  |
|  | density | √ | √ |  |  |  |  | √ |  |  | √ |  |  |
|  | rectangular fit |  |  |  | √ |  |  |  |  |  |  | √ | √ |
|  | border index |  |  | √ |  |  |  |  |  |  |  |  |  |
| T  E  X  T  U  R  E | GLCM  homogeneity | √ |  | √ | √ | √ | √ | √ | √ | √ |  | √ | √ |
|  | GLCM contrast |  | √ |  |  |  |  | √ |  |  |  |  |  |
|  | GLCM entropy | √ | √ |  | √ | √ | √ |  | √ | √ | √ | √ | √ |
|  | GLCM correlation | √ | √ |  |  |  |  |  |  |  |  |  | √ |
|  | GLCM  mean | √ |  | √ |  | √ | √ | √ | √ | √ |  | √ |  |
|  | GLDV entropy |  |  | √ | √ |  |  |  |  |  | √ |  | √ |
|  | GLDV contrast |  |  |  |  |  | √ |  | √ |  | √ |  |  |

**Supplementary Table 2.** Typical spectral characteristic for different land use types in this study. The imageries were extracted from the satellite imageries (Figure 1) to amplify the details of the different land use types (supported by SIGS (Worldview-2, 2013)). The figure was generated by H.X. using ArcMap 10.0 (<http://www.esrichina.com.cn/>. We have not used any map layer of the ESRI Company in this study).

| Farmland | 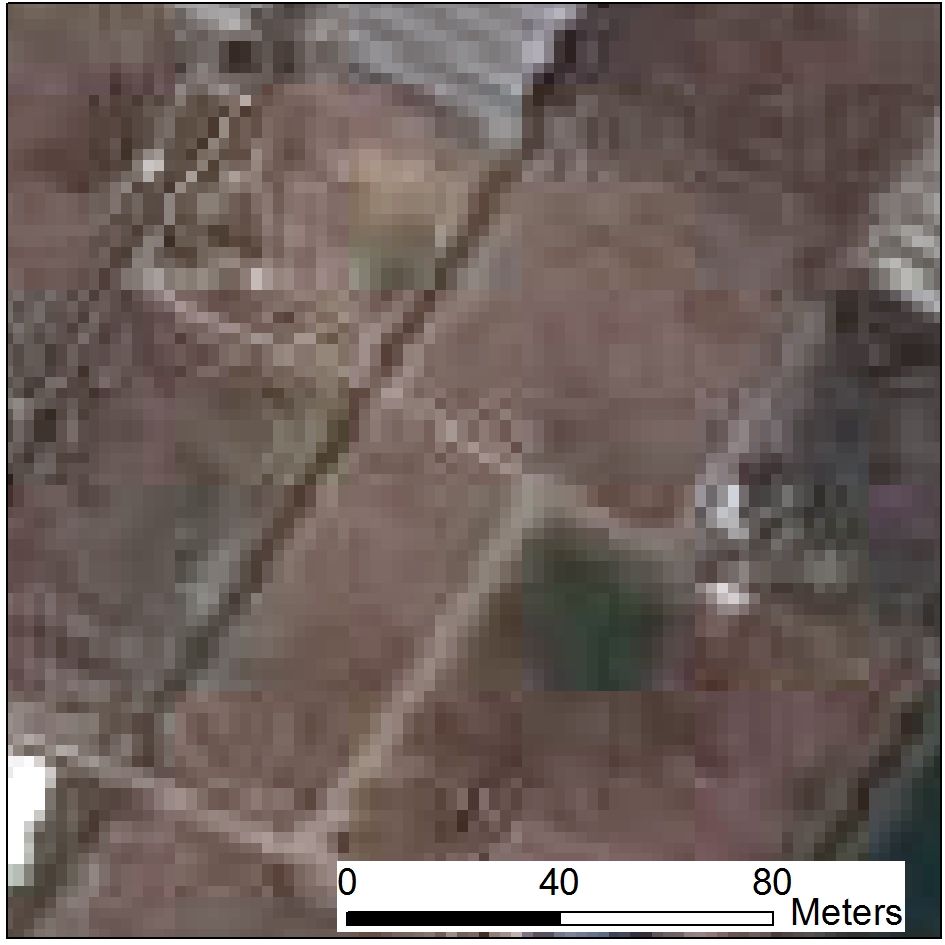 | 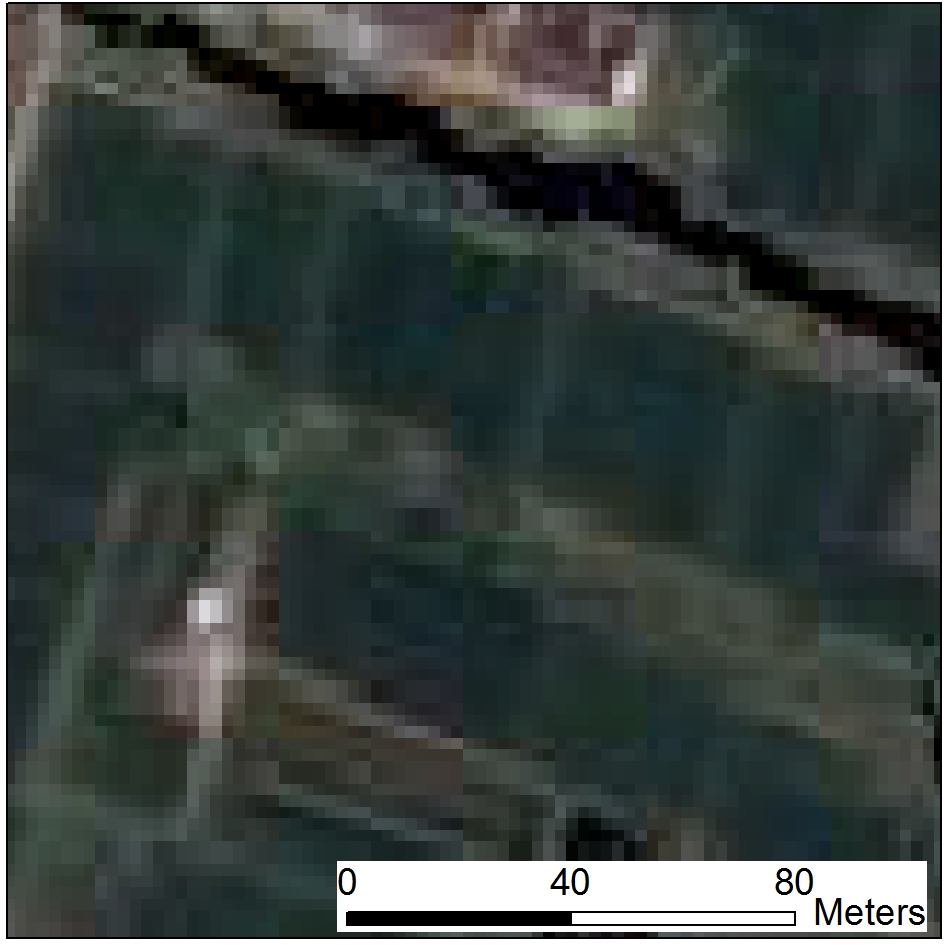 | 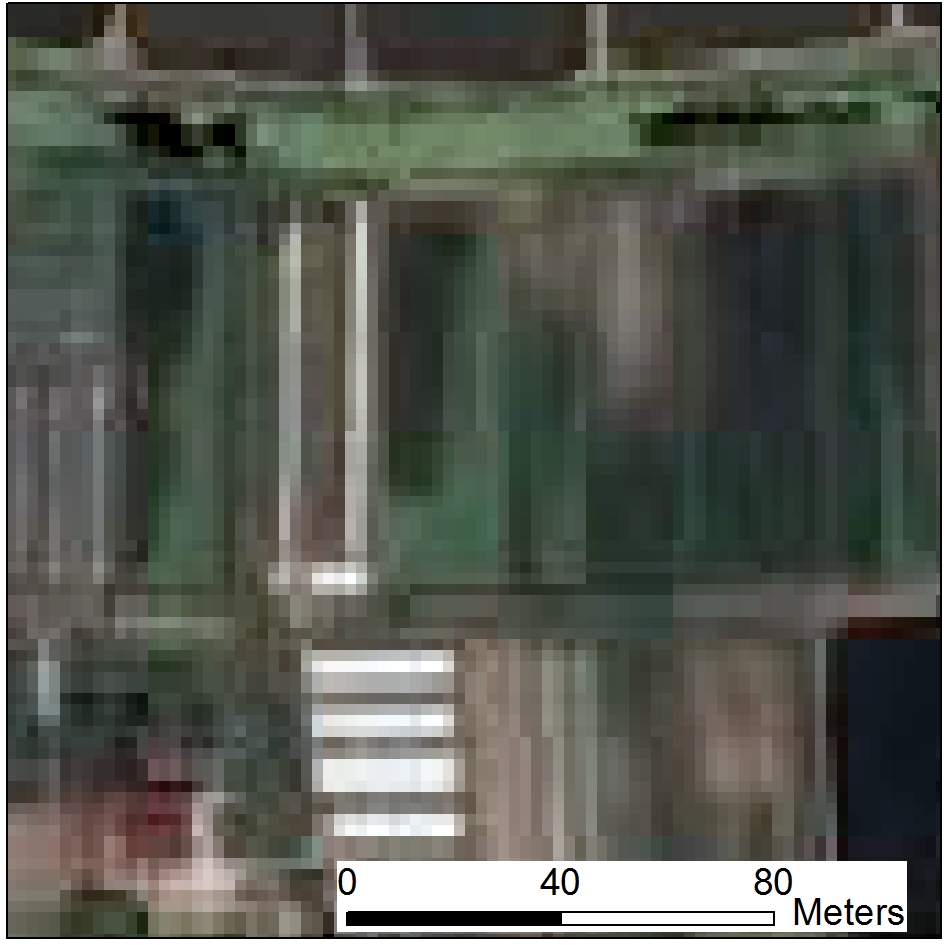 |
| --- | --- | --- | --- |
| Built-up | 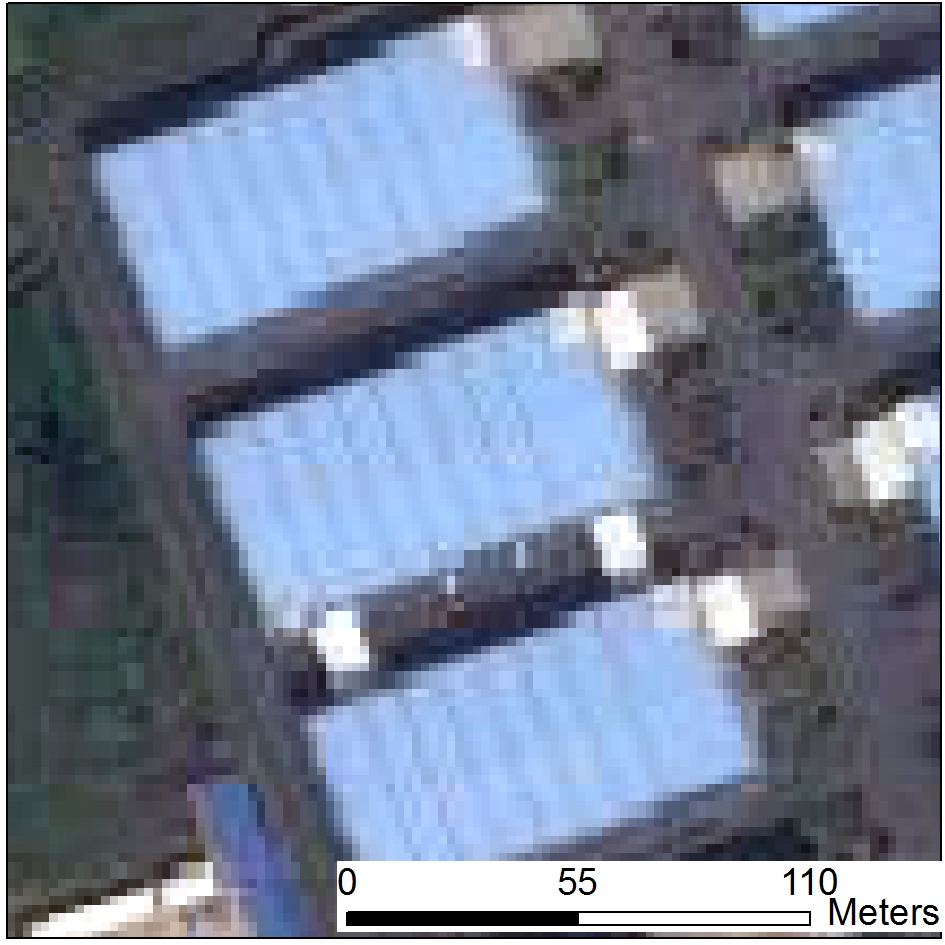 | 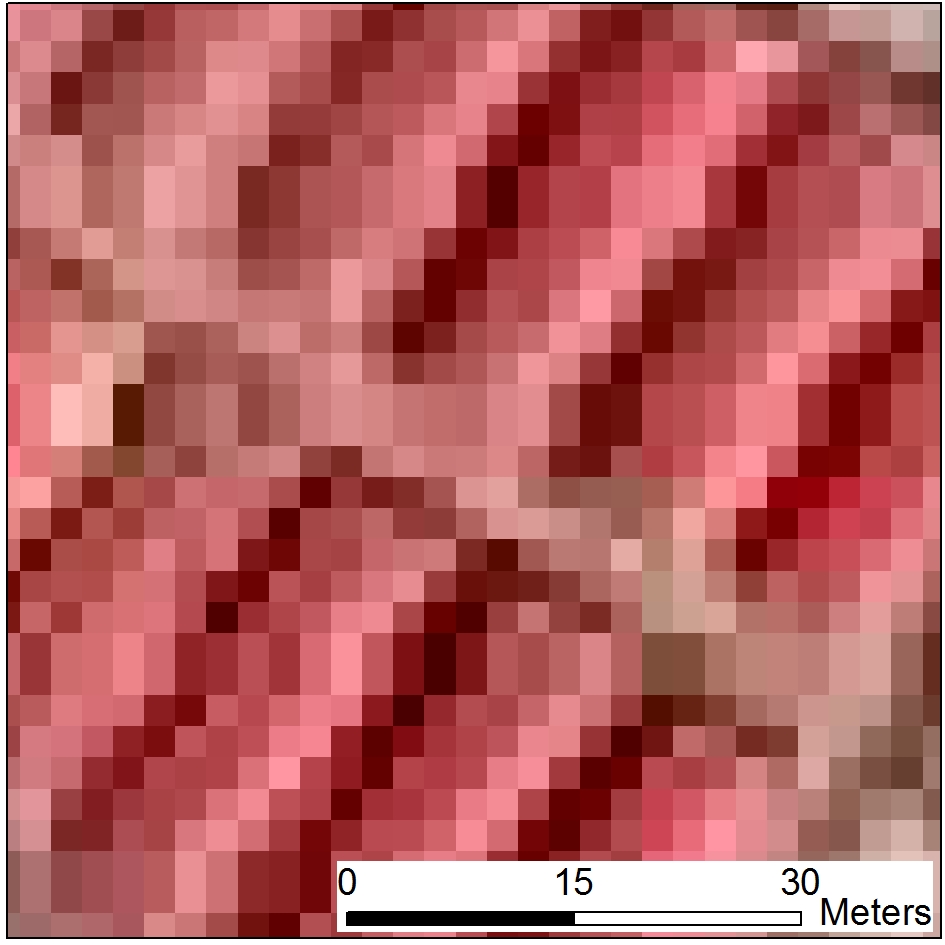 | 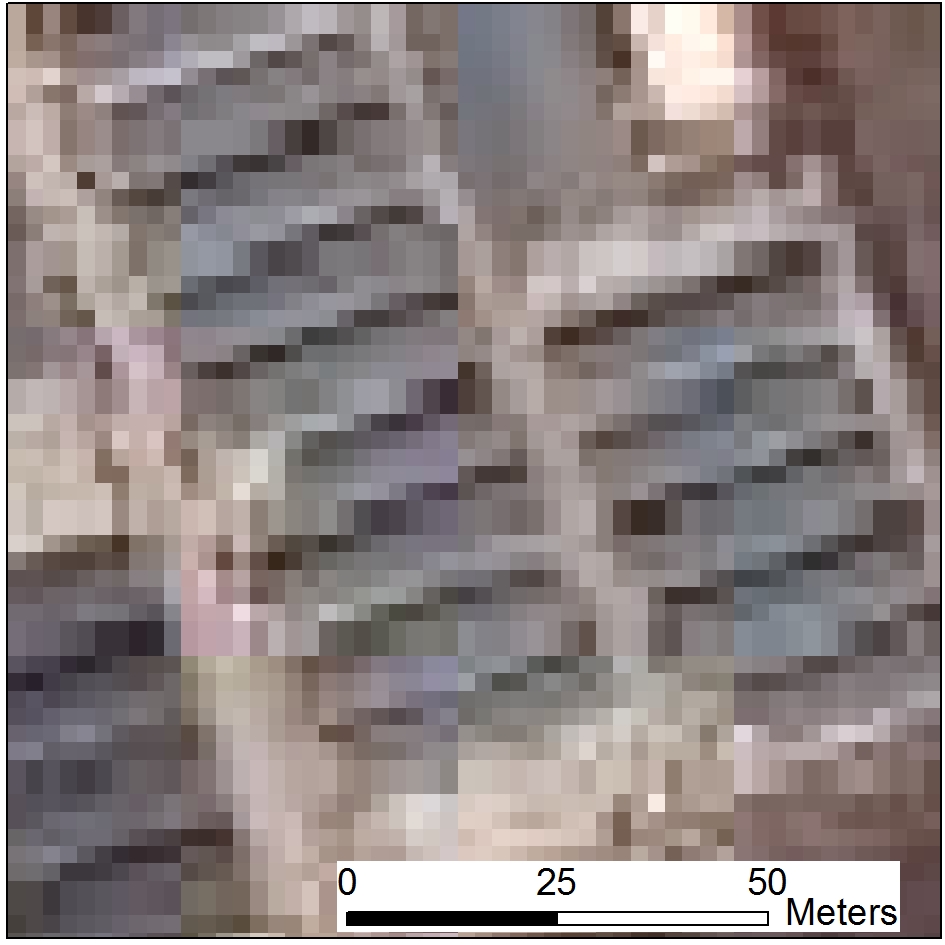 |
| Road | 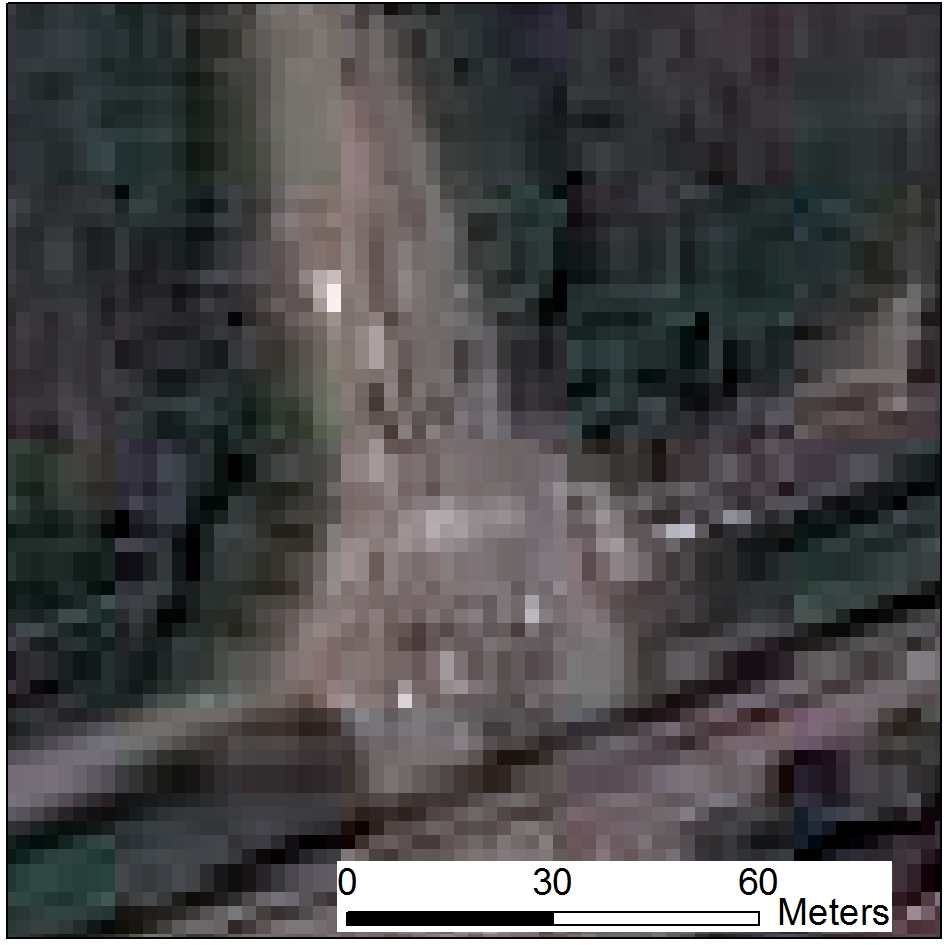 | 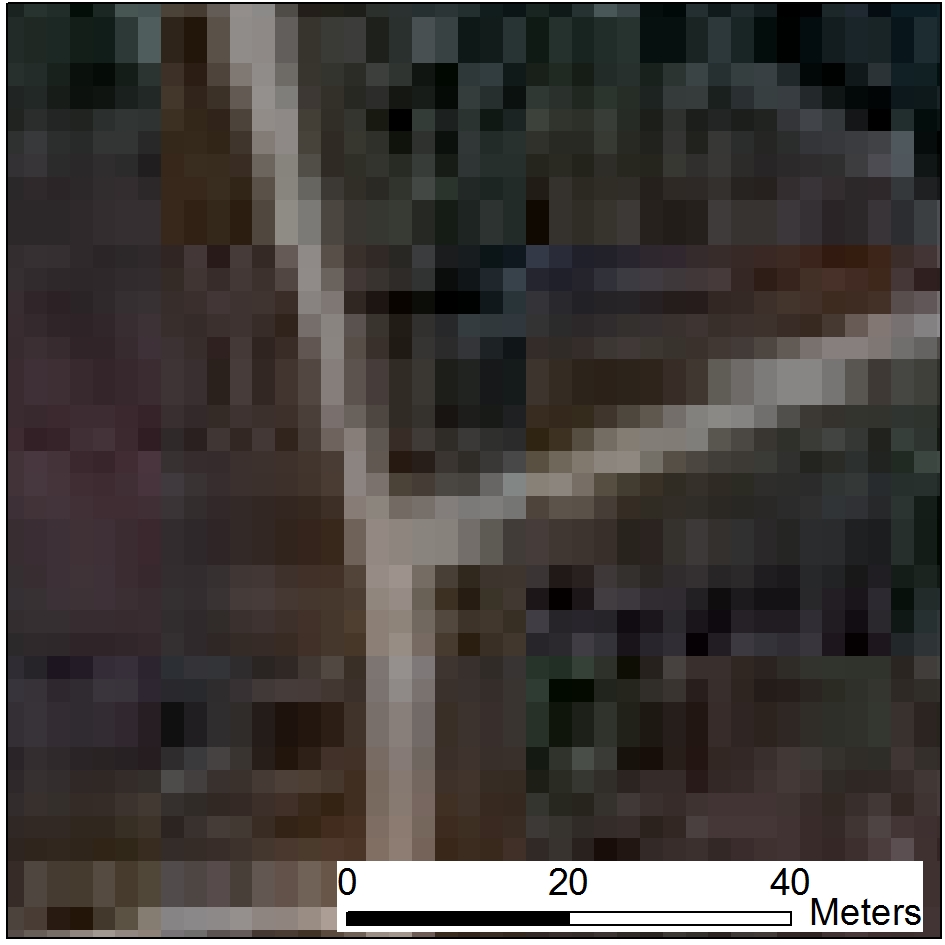 | 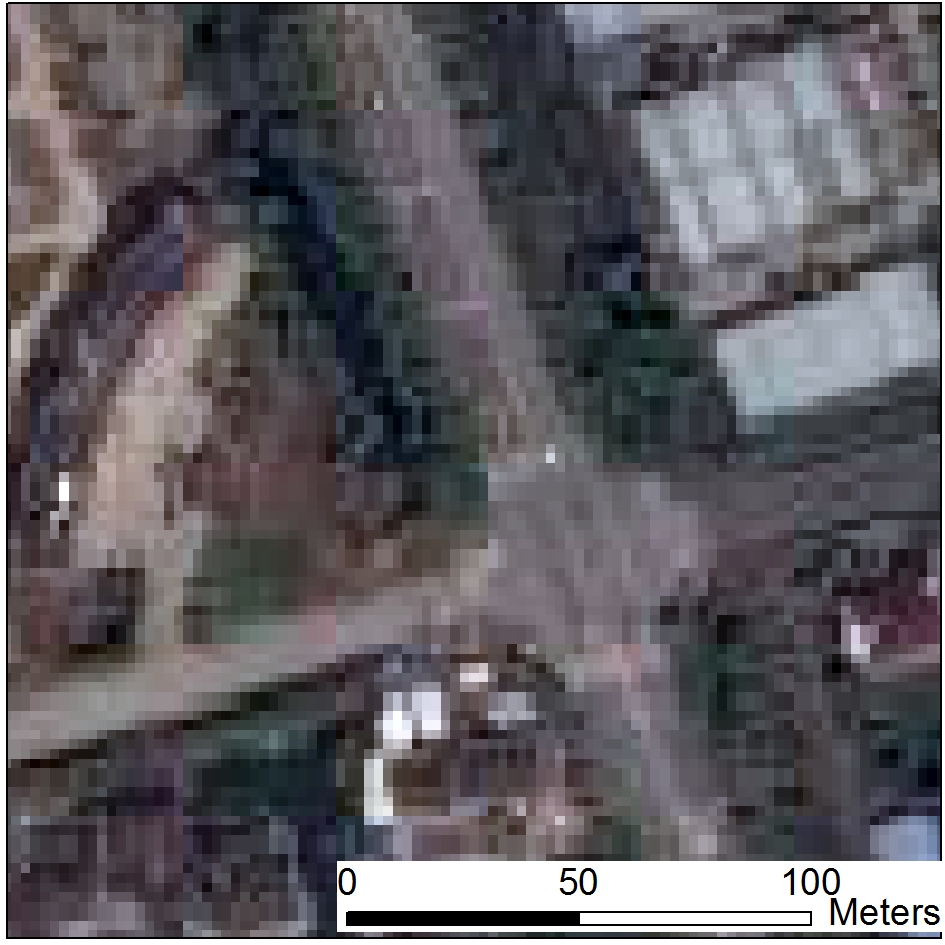 |
| Water | 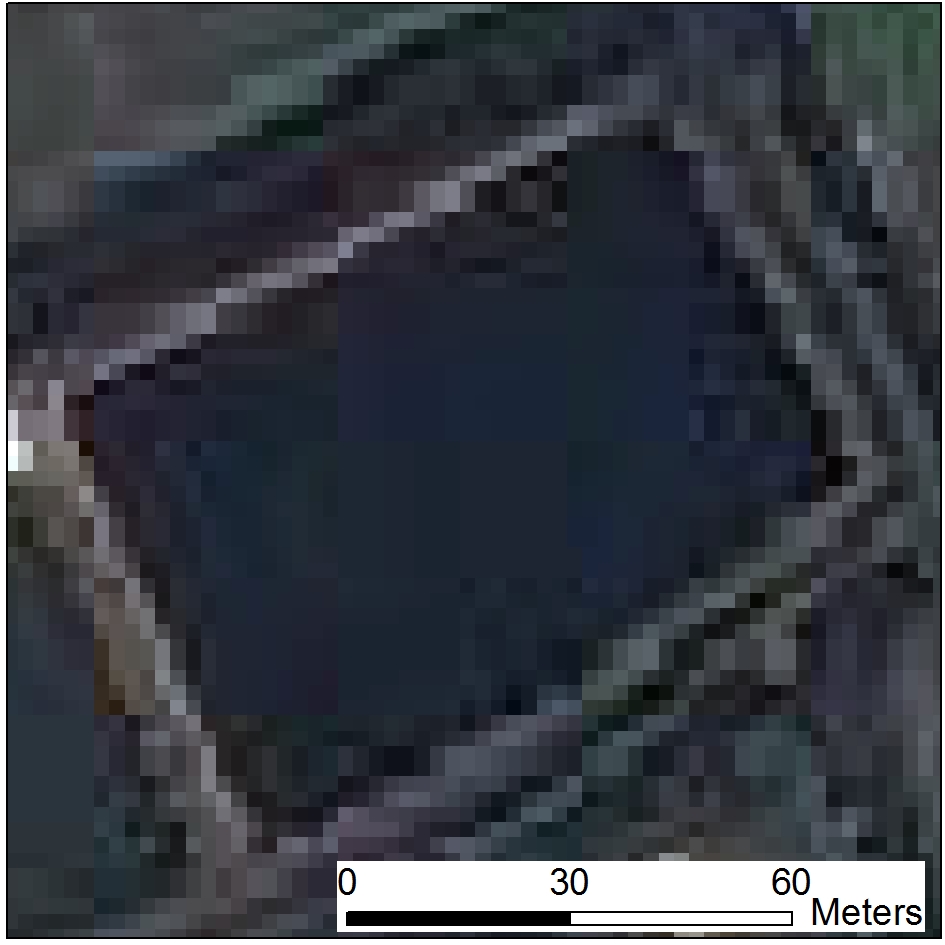 | 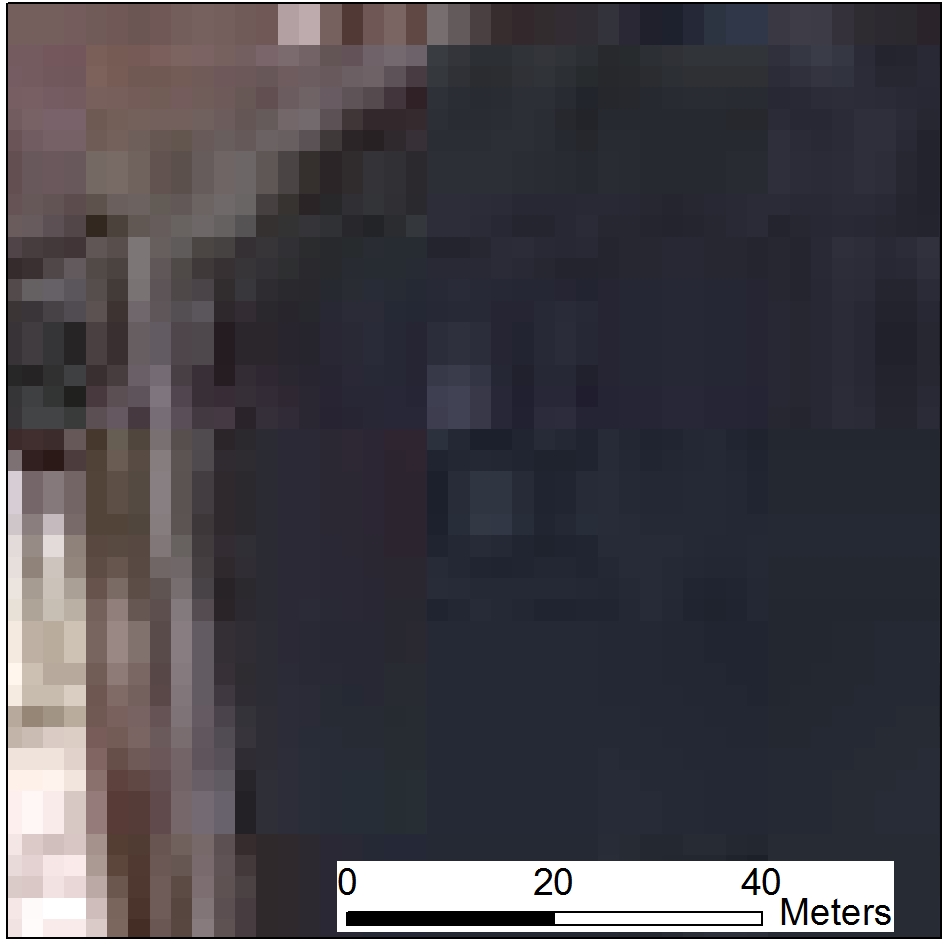 | 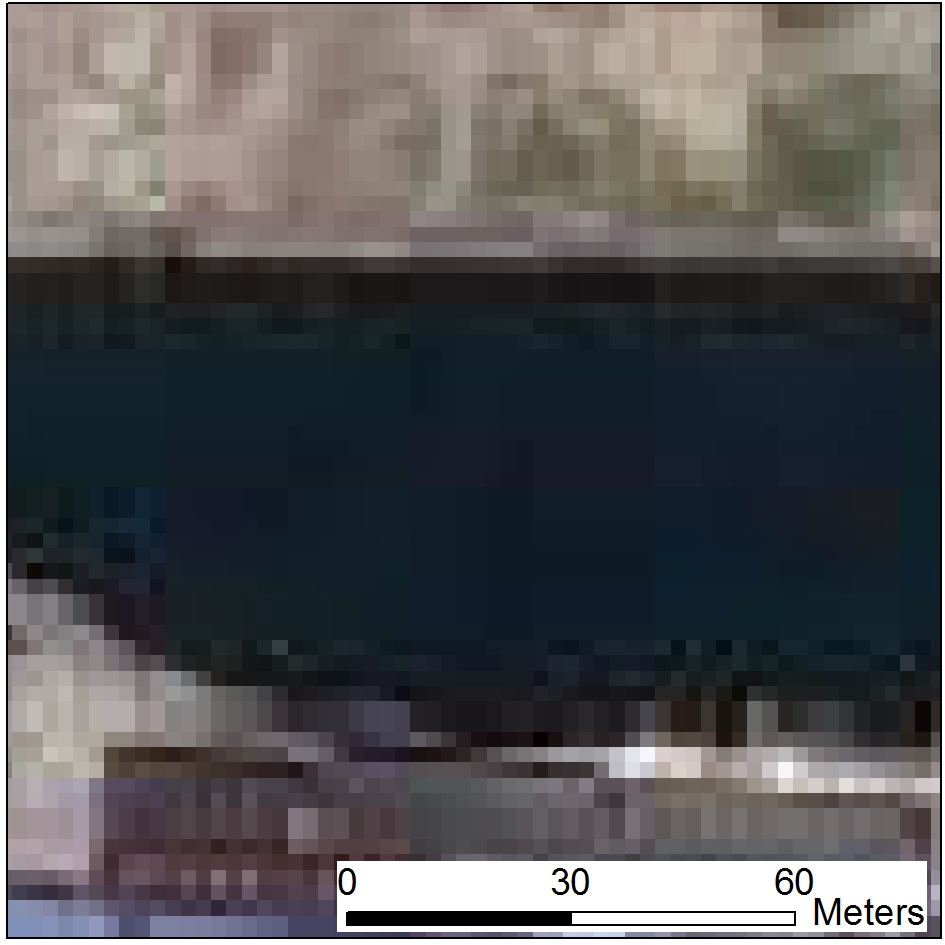 |
| Garden | 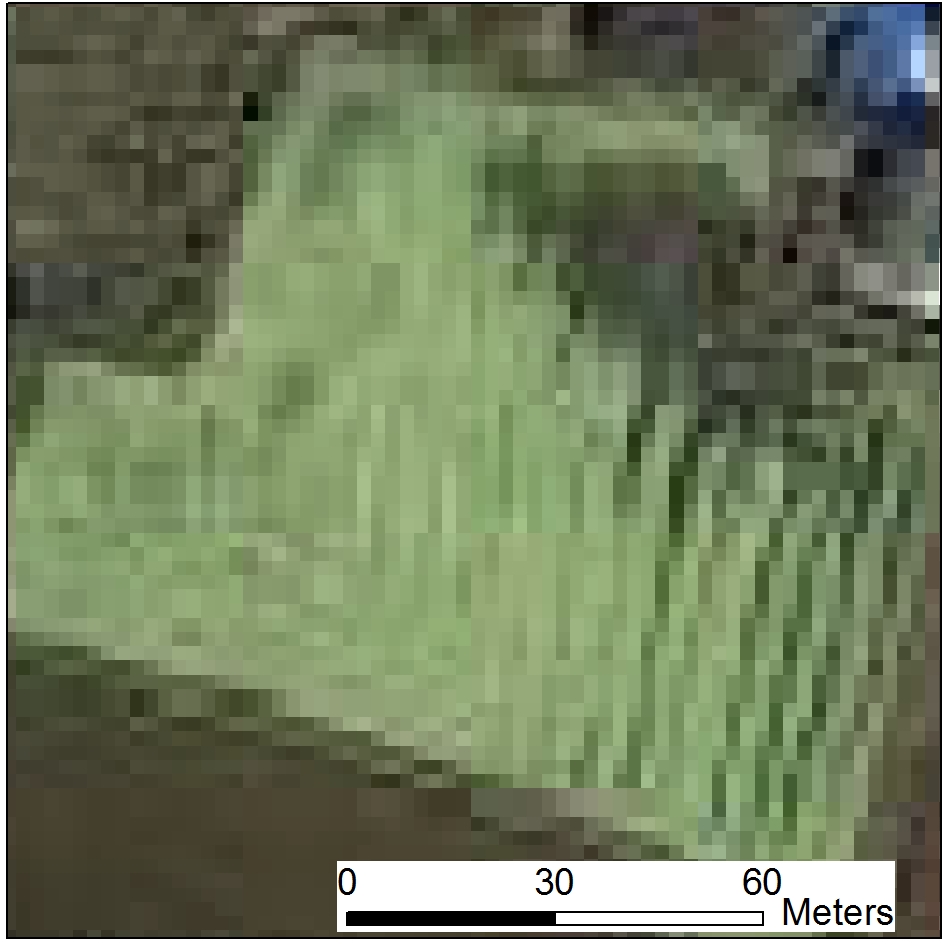 | 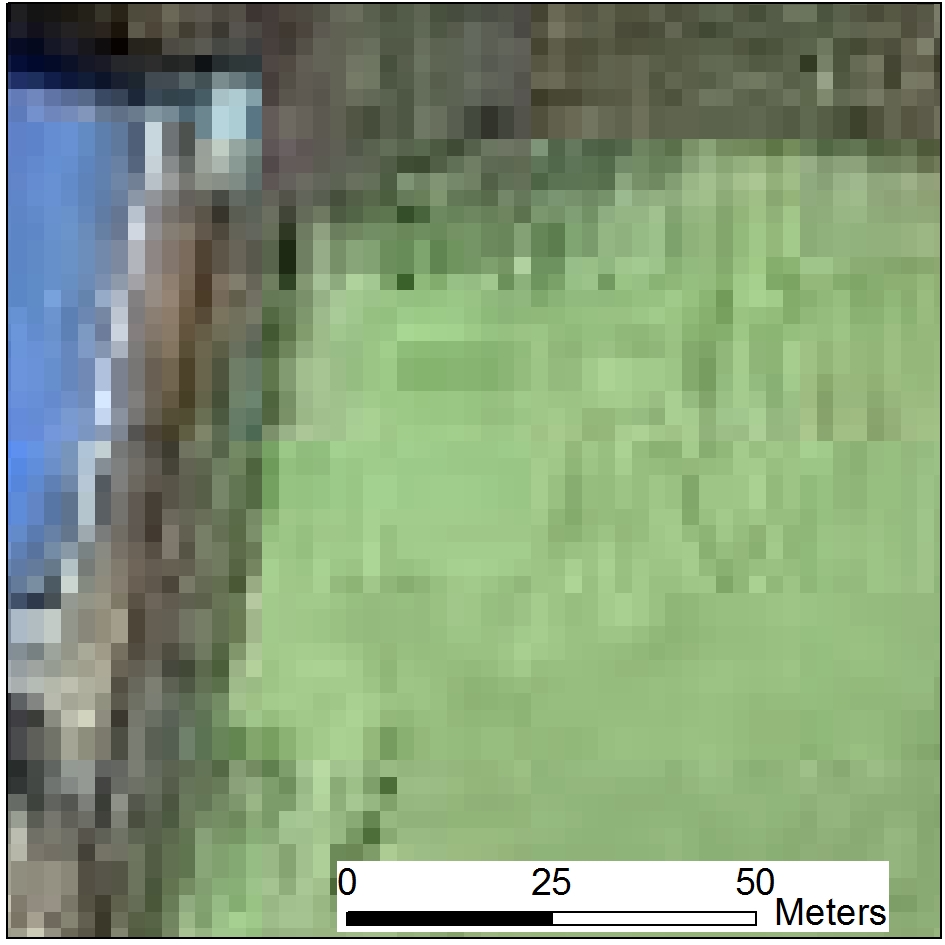 | 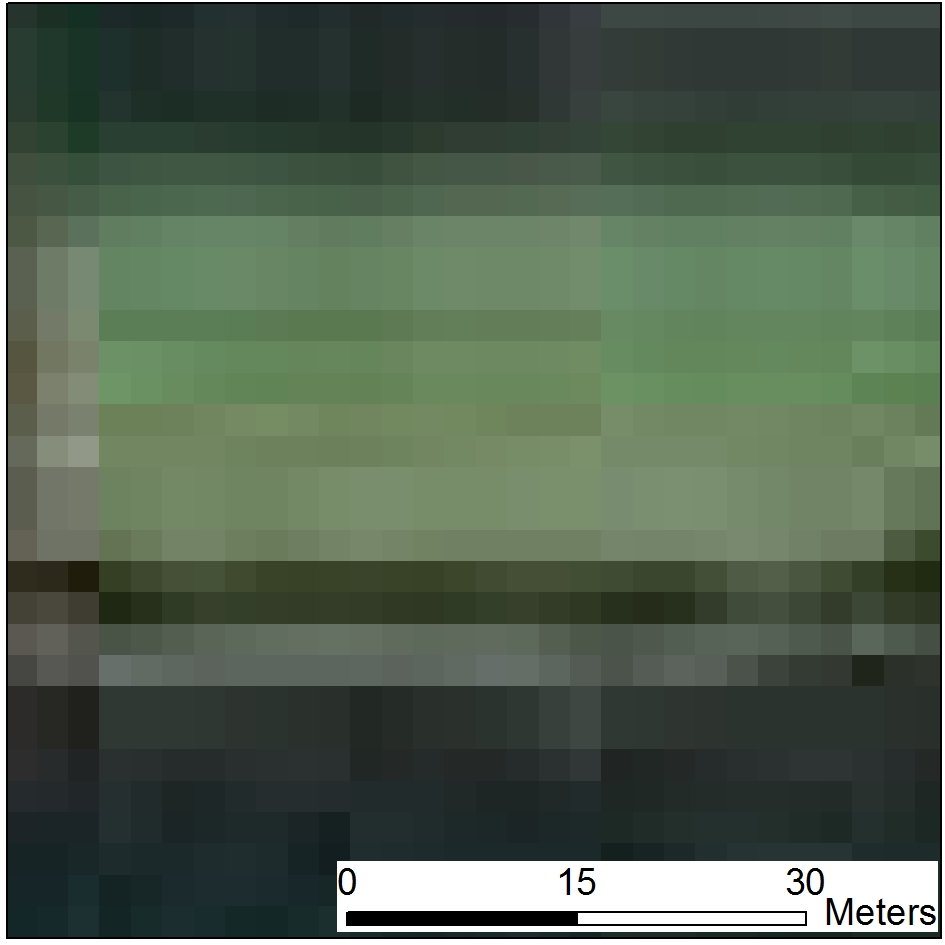 |
| Forest | 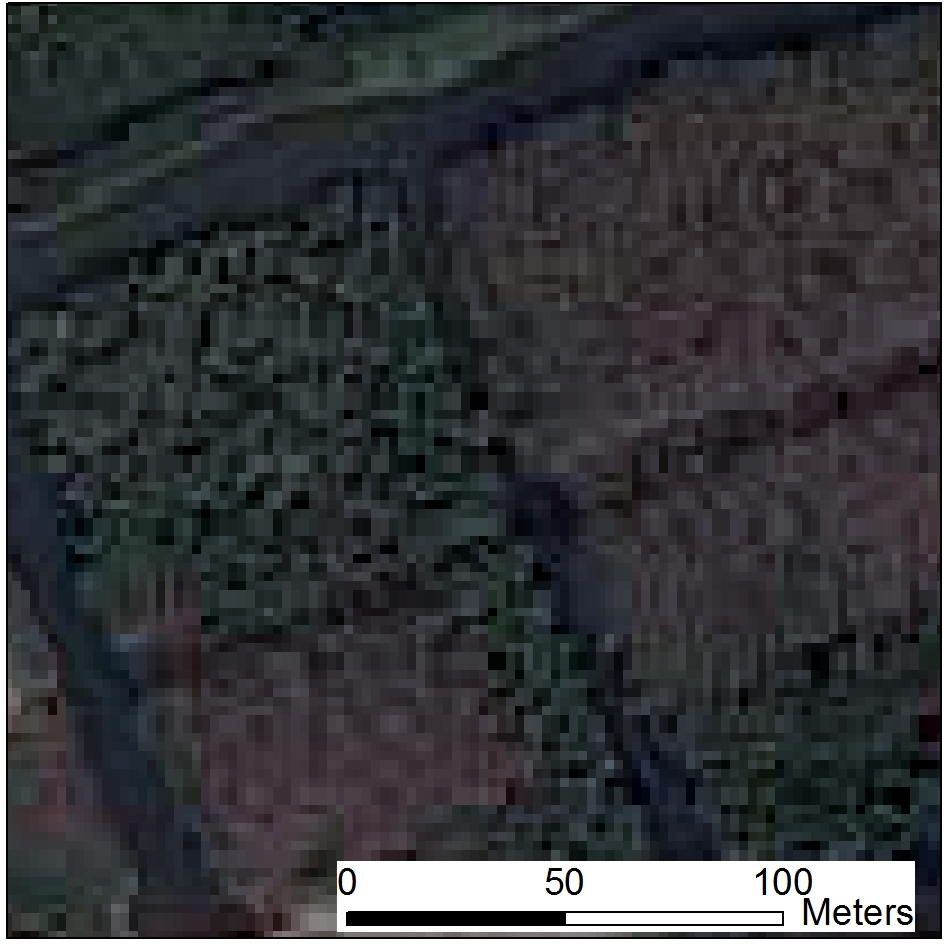 | 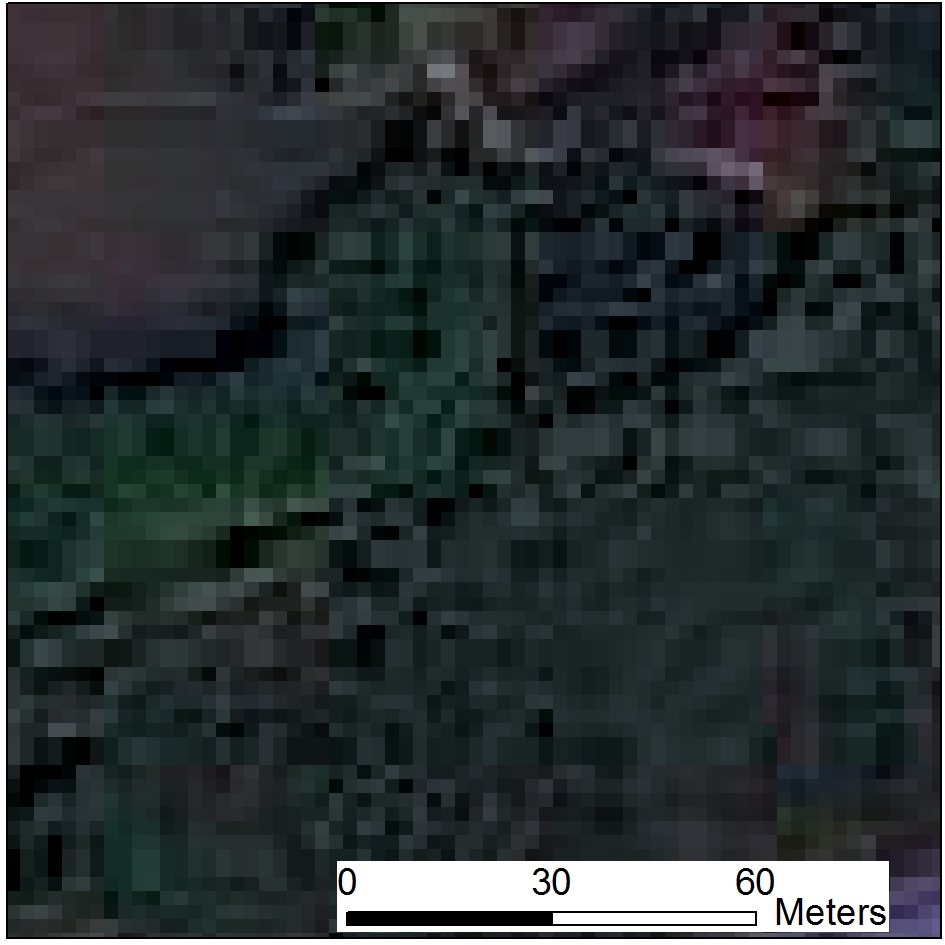 | 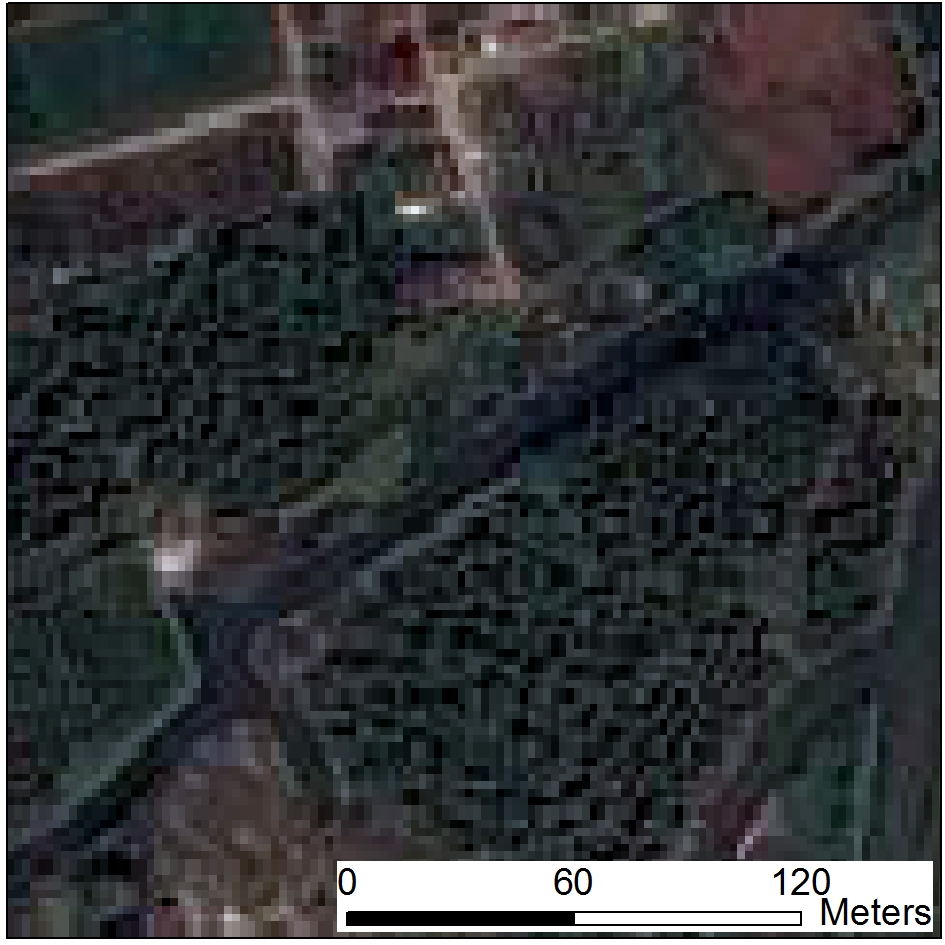 |
| Other | 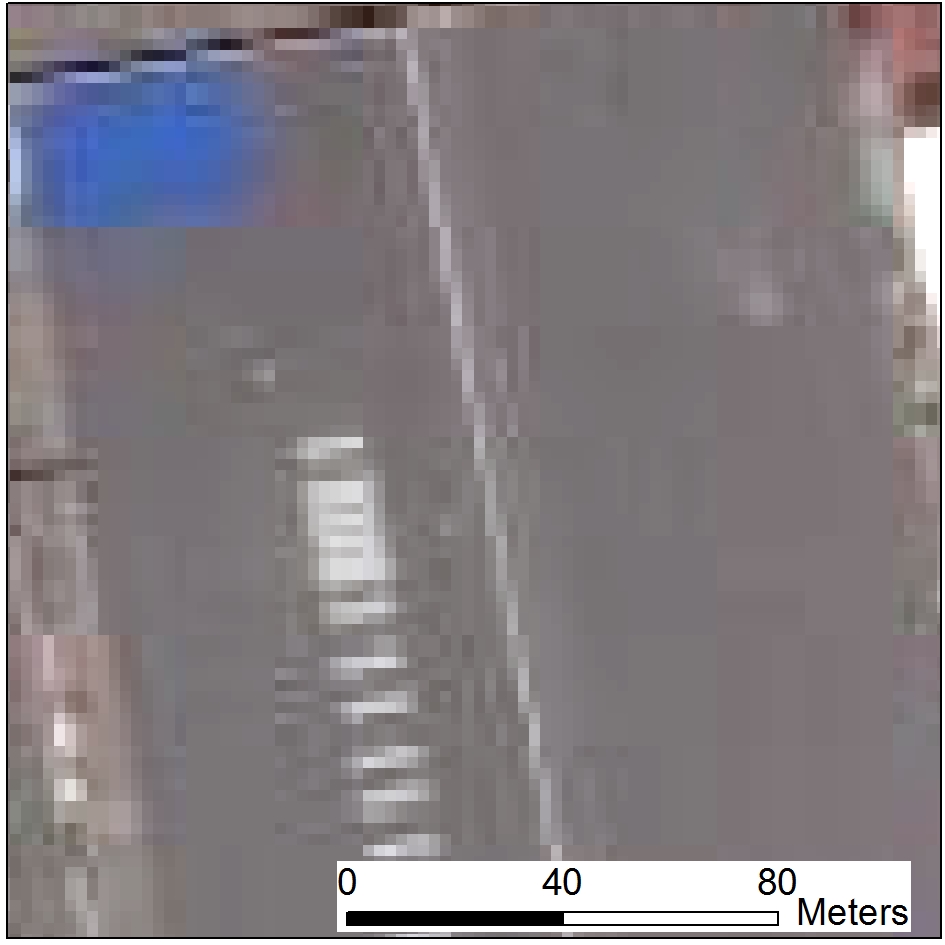 | 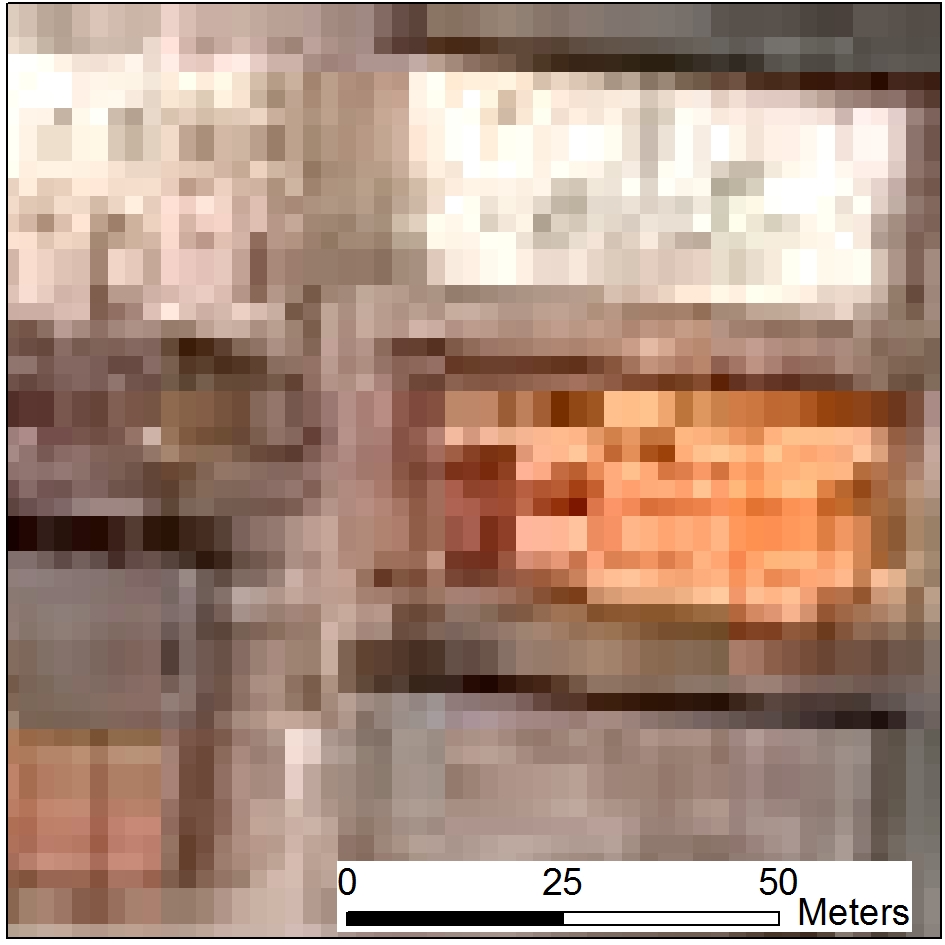 | 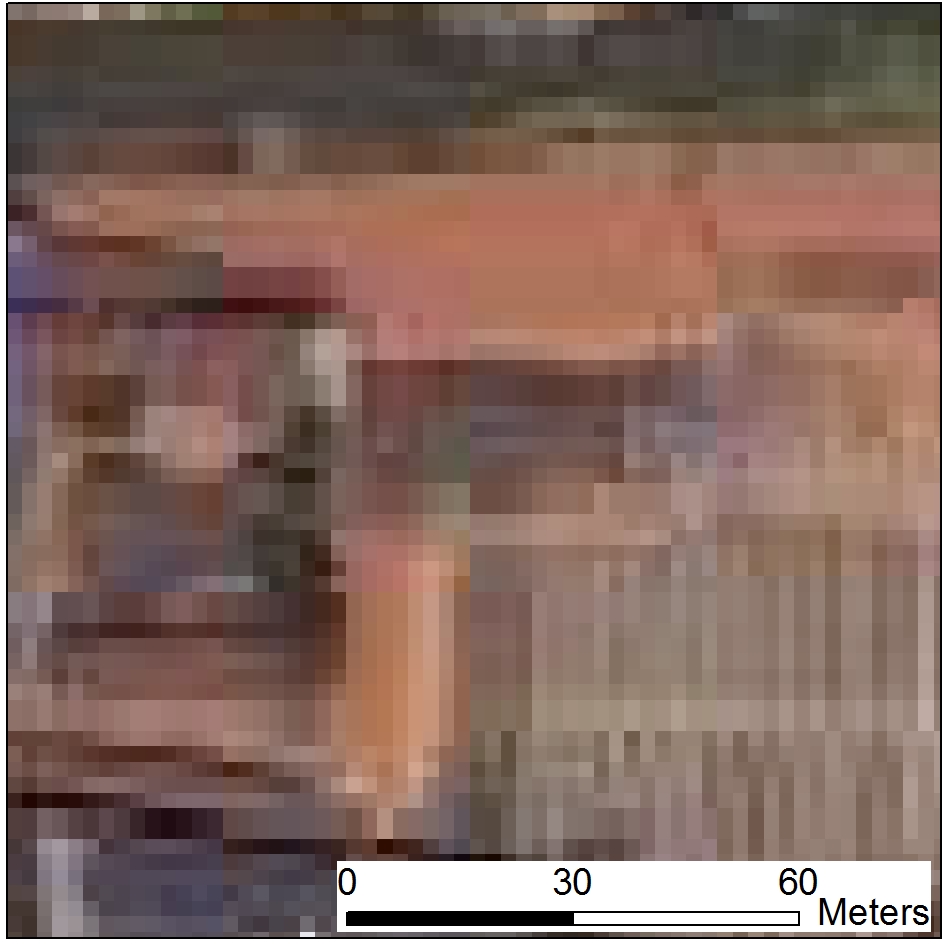 |

**Supplementary Table 3.** Classification results of 12 towns (the towns’ names are abbreviations and can be referred to Table 1). The land use types include farmland, built-up land, water body, road, garden land, forest land, and other land. Abbr. km2: square kilometers.

| Town | Farmland  area(km^2^) | Built-up  land(km^2^) | Road  (km^2^) | Water  body(km^2^) | Garden land(km^2^) | Forest  land(km^2^) | Other  land(km^2^) |
| --- | --- | --- | --- | --- | --- | --- | --- |
| MZ | 43.26 | 13.81 | 3.52 | 13.64 | 5.88 | 15.20 | 0.20 |
| GY | 42.42 | 10.82 | 3.29 | 9.80 | 1.06 | 7.67 | 0.06 |
| BH | 29.58 | 15.23 | 3.42 | 6.47 | 0.37 | 2.36 | 0.19 |
| LT | 33.80 | 14.18 | 3.97 | 25.71 | 1.05 | 14.81 | 0.13 |
| CS | 22.90 | 37.53 | 8.67 | 14.17 | 2.12 | 11.23 | 0.08 |
| DT | 15.14 | 14.22 | 3.53 | 7.71 | 9.49 | 0.43 | 0.12 |
| LG | 37.36 | 18.97 | 6.96 | 16.18 | 6.95 | 6.46 | 1.33 |
| JH | 26.78 | 25.49 | 3.60 | 12.93 | 0.68 | 3.23 | 0.11 |
| FC | 40.36 | 31.59 | 6.57 | 25.68 | 1.89 | 4.52 | 0.04 |
| ZX | 34.03 | 14.14 | 3.23 | 12.91 | 2.35 | 3.38 | 0.02 |
| YX | 32.95 | 13.49 | 4.47 | 9.00 | 1.68 | 10.89 | 0.05 |
| LX | 23.47 | 7.01 | 2.46 | 6.41 | 1.55 | 6.95 | 0.02 |

**Supplementary Table 4.** Confusion matrix of the land use classification in Miaozhen (MZ) Town. The unit of land use area was hectare (1 square kilometer = 100 hectare (hec.)). The UA and PA separately referred to user’s accuracy and production’s accuracy.

|  | | Truth (reference) data | | | | | | | | | | | | | | | | | |
| --- | --- | --- | --- | --- | --- | --- | --- | --- | --- | --- | --- | --- | --- | --- | --- | --- | --- | --- | --- |
|  | Farmland | | Built-up | | Road | | Water | | Garden | | Forest | | Other | | Sum (hec.) | | UA (%) | |  |
| Farmland | | 320.45 | | 5.39 | | 3.56 | | 12.52 | | 4.87 | | 5.96 | | 0.03 | | 352.78 | | 90.84 | |
| Built-up | | 2.36 | | 95.45 | | 2.20 | | 8.14 | | 1.12 | | 0.25 | | 0.08 | | 109.60 | | 87.09 | |
| Road | | 2.20 | | 5.16 | | 27.13 | | 6.97 | | 1.38 | | 0.31 | | 0.08 | | 43.22 | | 62.77 | |
| Water | | 1.18 | | 1.13 | | 1.35 | | 103.81 | | 1.22 | | 0.08 | | 0.00 | | 108.76 | | 95.45 | |
| Garden | | 22.26 | | 1.21 | | 1.49 | | 7.69 | | 45.00 | | 2.36 | | 0.01 | | 80.01 | | 56.24 | |
| Forest | | 9.05 | | 1.12 | | 1.30 | | 3.83 | | 7.34 | | 14.00 | | 0.00 | | 36.65 | | 38.20 | |
| Other | | 0.12 | | 0.11 | | 0.07 | | 0.08 | | 0.17 | | 0.13 | | 1.45 | | 2.12 | | 68.28 | |
| Sum (hec.) | | 357.62 | | 109.56 | | 37.10 | | 143.03 | | 61.10 | | 23.09 | | 1.65 | |  | |  | |
| PA (%) | | 89.61 | | 87.12 | | 73.12 | | 72.58 | | 73.65 | | 60.64 | | 87.70 | |  | |  | |
| Abbreviation: UA, user’s accuracy; PA, production’s accuracy;  hec., hectare (1 square kilometer = 100 hectare, 1 hectare= 10^4^ m^2^ );  Overall accuracy = 82.84%; Kappa Coefficient = 0.75. | | | | | | | | | | | | | | | | | | | |

**Supplementary Table 5.** The comparison of landscape pattern indices of the original PFs and the demarcated PFPAs. The indices include COHESION, MESH, LSI, and PAFRAC. And the SUM (area-weighted mean) values of each town in were also displayed.

| Town | **COHESION** | | **MESH** | | **LSI** | | **PAFRAC** | |
| --- | --- | --- | --- | --- | --- | --- | --- | --- |
|  | PFs | PFPAs | PFs | PFPAs | PFs | PFPAs | PFs | PFPAs |
| MZ | 98.12 | 99.92 | 2.16 | 174.02 | 153.30 | 56.36 | 1.26 | 1.23 |
| GY | 97.91 | 99.98 | 1.93 | 788.98 | 170.21 | 48.75 | 1.23 | 1.24 |
| BH | 98.15 | 99.94 | 2.37 | 262.91 | 89.61 | 29.51 | 1.13 | 1.16 |
| LT | 98.28 | 99.82 | 4.74 | 40.28 | 86.25 | 36.02 | 1.18 | 1.16 |
| CS | 97.84 | 99.74 | 1.70 | 18.72 | 114.95 | 45.68 | 1.17 | 1.19 |
| DT | 97.79 | 99.74 | 1.41 | 20.25 | 92.44 | 42.44 | 1.18 | 1.21 |
| LG | 98.54 | 99.84 | 16.63 | 117.82 | 86.61 | 32.04 | 1.14 | 1.18 |
| JH | 98.14 | 99.84 | 4.17 | 47.41 | 87.39 | 36.67 | 1.14 | 1.16 |
| FC | 98.16 | 99.85 | 3.37 | 50.84 | 127.30 | 55.19 | 1.21 | 1.21 |
| ZX | 98.26 | 99.83 | 6.74 | 40.86 | 81.30 | 38.65 | 1.13 | 1.18 |
| YX | 98.22 | 99.91 | 3.19 | 128.41 | 90.43 | 30.83 | 1.17 | 1.16 |
| LX | 98.30 | 99.92 | 3.47 | 163.93 | 80.13 | 33.39 | 1.20 | 1.22 |
| **SUM** | 98.15 | 99.86 | 4.56 | 147.04 | 107.92 | 41.63 | 1.18 | 1.19 |

**Supplementary Table 6.** The correlation of the dual-threshold matrix between each town (Supplementary Figure 4) and the bold values indicate relatively stronger correlations between the two corresponding cities (mainly > 0.98). MZ, GY, BH, LT, ZX, YX, and LX only have relative strong correlations with each other and could be classified into one group. Similar rules could be found for CS, DT, LG, JH, and FC.

| Town | MZ | GY | BH | LT | CS | DT | LG | | JH | FC | ZX | YX | LX |
| --- | --- | --- | --- | --- | --- | --- | --- | --- | --- | --- | --- | --- | --- |
| MZ | - | **0.990** | **0.996** | **0.991** | 0.947 | 0.945 | 0.890 | | 0.971 | 0.970 | **0.992** | **0.993** | **0.997** |
| GY | **0.990** | - | **0.991** | **0.979** | 0.942 | 0.932 | 0.880 | | 0.973 | 0.961 | **0.985** | **0.982** | **0.984** |
| BH | **0.996** | **0.991** | - | **0.985** | 0.929 | 0.923 | 0.859 | | 0.973 | 0.960 | **0.987** | **0.996** | **0.994** |
| LT | **0.991** | **0.979** | **0.985** | - | 0.958 | 0.953 | 0.913 | | 0.938 | 0.958 | **0.997** | 0.969 | **0.991** |
| CS | 0.947 | 0.942 | 0.929 | 0.958 | - | **0.995** | **0.981** | | **0.985** | **0.981** | 0.969 | 0.924 | 0.940 |
| DT | 0.945 | 0.932 | 0.923 | 0.953 | **0.995** | - | **0.983** | | **0.983** | **0.980** | 0.963 | 0.920 | 0.939 |
| LG | 0.890 | 0.880 | 0.859 | 0.913 | **0.981** | **0.983** | - | | **0.980** | **0.979** | 0.922 | 0.859 | 0.883 |
| JH | 0.971 | 0.973 | 0.973 | 0.938 | **0.985** | **0.983** | **0.980** | | - | **0.998** | 0.963 | 0.972 | 0.953 |
| FC | 0.970 | 0.961 | 0.960 | 0.958 | **0.981** | **0.980** | **0.979** | | **0.998** | - | 0.905 | 0.957 | 0.936 |
| ZX | **0.992** | **0.985** | **0.987** | **0.997** | 0.969 | 0.963 | 0.922 | | 0.963 | 0.905 | - | **0.988** | **0.991** |
| YX | **0.993** | **0.982** | **0.996** | 0.969 | 0.924 | 0.920 | 0.859 | | 0.972 | 0.957 | **0.988** | - | **0.996** |
| LX | **0.997** | **0.984** | **0.994** | **0.991** | 0.940 | 0.939 | 0.883 | | 0.953 | 0.936 | **0.991** | **0.996** | - |
| The correlation values between different two matrixes were measured by coefficient r using Matlab. Where A,B is the matrix, 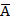,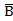 is the mean values of matrix, and m,n is the row and column number. | | | | | | | |  | | | | | |

**Supplementary Approach**

## A. OBIA: Image segmentation and feature selection

To extract farmland information from the WV2 images, the flowing workflow was implemented: (**A1**) segment images at multiple scales, (**A2**) use the stratified random scheme to obtain reference data and use the Gini index to select appropriate features, (**A3**) classify the segmented images based on the selected features using a random forest (RF) classifier, and (**A4**) evaluate the accuracy using an area-based method

### A1. Image segmentation at multiple scales

We used the eCognition 8.7 software (<http://www.ecognition.com/>) to implement the image segmentation and to create different image objects. The eCognition developer’s proprietary multiresolution segmentation (MRS) is known as one of the most successful segmentation algorithms, which is a bottom-up region-growing and region-merging technique ^[2]^. Smaller image objects are merged into bigger objects based on the overall heterogeneity (*f*) of adjacent objects, where the parameter *f* is defined as the combined weighted value of the spectral and shape heterogeneities ^[3]^. The MRS procedure begins with single-pixel objects in images and repeatedly conducts a clustering process based on minimal heterogeneity. The procedure stops once the smallest growth exceeds the threshold defined by the scale parameter ^[3]^. Therefore, the scale parameter directly determines the segmentation results and defines the maximum standard of the homogeneity criterion ^[2]^. A larger value of the scale parameter can fuse a greater number of objects and yield larger objects. We selected a scale of about 80 for the image segmentation in our study area, where the optimal scale was strongly associated with the average size of the actual objects by manual interpretation ^[2] [4]^.

### A2 Reference data and feature selection

After image segmentation by eCognition 8.7, the basic image units become irregular polygonal objects formed by homogeneous pixels, and a reliable classification strategy should be applied on the segmentation results to extract land use/cover information ^[2]^. The classification accuracy is highly dependent on the selection of training and validation samples ^[5]^. The training samples are used for calibrating and testing the classification algorithm, and the validation samples are utilized for verifying the results. Traditionally, the training samples were manually photointerpreted from both the image data itself and imageries with higher spatial resolution, such as Google Earth. The polygon-based sample unit has been proven effective in OBIA, and the attribute label of the polygon object is determined by the majority class (>60%) of the polygon ^[6]^. Here, stratified random sampling was used to obtain reference data for training and assessment ^[7]^. Stratified random sampling, need some prior knowledge about the proportion of different land use types, which can ensure the unbiased sample selection and appropriate sample numbers in each class ^[6]^. The relevant land use proportion data were from the statistical yearbooks of each town in Shanghai (<http://www.stats-sh.gov.cn/> ). For each town, the reference data area was about 20-30% of total area, and about 3/4 of the data for training and 1/4 for assessment.

The selection of features was based on previous studies ^[2], [8]^, which included spectral characteristics, shape features and textural features. To reduce redundancy and intercorrelation among the 24 types of optional features, it was necessary to select an optimal feature subset. A common splitting strategy is the Gini index ^[9]^, which is defined as:

| $\text{G(}t)=1-\sum_{k=1}^{Q} p^{2}(k\vert t)$ | …………………………….**(1)** |
| --- | --- |

where *t* is a node, *p*(*k*|*t*) (*k* = 1,2,…,*Q*) is the estimated class probabilities, and *Q* is the number of classes. If all the objects in node *t* belong to the same class, the Gini index is zero; when the different class sizes at node *t* are equal, the index is one. In effect, the Gini index is a measure of heterogeneity ^[10]^. The Gini index was used at each node of the decision trees, and the highest change determined the variable importance of different features ^[10], [11]^. The spectral features almost have a greater variable importance, and only 5-6 shape (sorted by variable importance) and texture features were selected for the convenience of calculation ^[2]^, ^[10], [11]^ (variable importance threshold ≈ 1/4-1/3 * largest variable importance).

The optional features conclude: (1) spectral feature measures: 3 means, 3 standard deviations, max difference and brightness of image bands; (2) shape features: area, compactness, density, roundness, main direction, rectangular fit, elliptic fit, asymmetry, border index, and shape index; (3) texture features: GLCM (Gray-Level Co-occurrence Matrix) homogeneity, GLCM contrast, GLCM dissimilarity, GLCM entropy, GLCM std. dev., GLCM correlation, GLCM ang. 2nd moment, GLCM mean, GLDV (Gray-Level Difference Vector) ang. 2nd moment, GLDV entropy, and GLDV contrast. The GLCM and GLDV were calculated based on pixels in each object. They were used for each segmentation layer and in the feature selection algorithm (For more details, see eCognition documentation ^[1]^).

### A3 Random forest (RF) classification

The RF algorithm is a robust machine-learning classifier that uses a random iterative method to establish a forest from mutually independent decision trees (DTs) ^[12], [13]^. The basic classifier for an RF is the classification and regression tree, which is a type of DT based on the relative importance to classification and regression. After obtaining the forest from the training set, we let each DT in the forest make a judgment about the unlabeled sample according to the features determined by the previous Gini index. Then the unlabeled sample was predicted as the category that was voted for most frequently in all suitable DTs.

The basic steps used in RFs are: (1) each RF tree is grown on a bootstrap or bagging sample of the training set, (2) when growing a tree, at each node, *n* variables are selected randomly out of the *N* available (*N* is the number of the imported features), (3) usually, *n* << *N*, it is suggested to start with *n* = log_2_(*N*) + 1 or *n* =
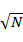
 before decreasing and increasing *n* until the minimum error is obtained for the out-of-the-bag dataset. At each node, only the variable that provides the best spilt is used out of the *n* selected ^[9]^.

The RF classifier only requires two parameters to generate a prediction model: the number of classification trees (*k*) and the number of the prediction variables (*n*) at each node. According to related research ^[9], [14]^, large tree numbers (*k*) and small split variables (*n*) can reduce generalization errors and correlation between trees. Our RF models used single randomly split variable and 479 univariate DTs ^[2], [14]^ for the WV2 classification. The classification results were stored using a polygon shapefile (an ESRI data format describing the spatial data).

### A4 Accuracy assessment

Different from pixel-based accuracy assessment methods, we applied an area-based method for quantitative accuracy assessment. This method evaluates the classification accuracy by the feature extent and spatial distribution ^[15]^. It can also facilitate the production of a confusion matrix ^[16]^, which could be used to calculate the User’s Accuracy (UA) and Producer’s Accuracy (PA) for each class. The area-based method conduct the overlap analysis based on the extracted (classified) and reference vector data set. The overall thematic accuracy (TA) is calculated by dividing the overlapped area of the two data sets (intersection, ∩) by the area of their union set (union, ∪):

$TA=\frac{Area(Ext\text{∩}Ref)}{Area(Ext\text{∪}Ref)}\text{ }\left|\text{E∪R}\right|\text{=}\left|\text{E∩R}\right|\text{+}\left|\text{E∩¬R}\right|\text{+}\left|\text{R∩¬E}\right|$ **(2)**

$UA=\frac{Area(Ext\text{∩}Ref)}{Area(Ext)}$ $PA=\frac{Area(Ext\text{∩}Ref)}{Area(Ref)}$ **(3)**

where *Ext* is the extracted vector data from the imageries and *Ref* is the reference vector data. To advance our understanding of classification accuracy, we calculated the commission and the omission errors to conduct a Kappa analysis ^[17]^. The Kappa coefficient (KC) and overall analysis (OA) can be calculated from confusion matrices produced with cross-validation training samples or independent validation samples.

## B. LESA/Land evaluation

Here, the method was applied to the image classification results and could quantify the farmland quality. The land evaluation unit in this study was the each complete farmland patch, instead of tradition evaluation unit based on the grid cell (unified shape and size).

### B1 Land evaluation

The original LE component contained 6 factors (the effective soil thickness, organic matter content, surface soil texture, soil PH, topographic slope, and conditions of surface rock outcrops). China has established a gradation and valuation system for agricultural land and the natural grade of farmland in this system reflects the natural production condition, which is essentially equal to the LE component of LESA. We obtained the natural gradation data of farmland (2009) from SIGS, which were classified into 15 levels where level 1 represents the highest quality (for further details refer to Regulations for gradation on agriculture land quality ^[18]^ and Regulations for classification on agriculture land ^[19]^). Although the data concerning the distribution and area of farmland differed from 2013, the interpretations were considered largely reliable and valid ^[20]^. Therefore, we used a weighted approximation algorithm to obtain the natural gradation value of farmland in 2013:

We made the rule that, (1) if the farmland patch in 2013 intersected with only one patches in 2009, the attribute equal to attributes in 2009 (2) if the 2013 patch intersected with k patches in 2009, the attribute equal to the area-weighted average of these k patches (3) if the 2013 patch intersected with no patches in 2009, the attribute equals to the distance-weighted average value of neighboring 4 patches:

$LE^{j}_{2013}=\left\{\begin{matrix} NG^{i}_{2009}\text{ } \\ \sum\omega^{i}_{area}*NG^{i}_{2009}\text{ }\omega^{ij}_{area}=area_{ij}/\sum area_{ij}\text{ } \\ \sum\omega^{i}_{dis}*NG^{i}_{2009}\text{ }\omega^{ij}_{dis}=(1/dis_{ij})/\sum(1/dis_{ij})\text{ } \end{matrix}\right.$ **(4)**

Where area_i_ is the intersect area between the target farmland patch j (2013) and the pacth i (2009), dis_ij_ is the distance between the centroid of patch I and j, NG^i^_2009_ is the natural gradation value of patch i.

The evaluation unit was independent farmland vector patch. The LE score, directly calculated from the natural gradation, was normalized to 0-10 where 10 has the highest quality grade (Supplementary Figure 2-1).

### B2 Site assessment

The SA component is mainly a reflection of external pressures. Based on related research and the actual situation in Shanghai, we selected four factors: the distance to built-up land, density of road networks, density of irrigation water, and distance to the farmers’ market ^[21]^. The first three factors were calculated based on the classification results where water bodies were similar to the irrigation water. Data on farmers’ markets were provided by the SIGS and converted to point data. The evaluation unit was independent farmland vector patch, and the score for the four factors was based on the distance decay function. For the benefit of the calculation, scores of each factor were normalized to 0-10 where 10 has the highest quality grade. The SA results were the weighted summation of the four factors and the weight of each was 0.25 (Supplementary Figure 2-2).

The calculation of the four factors were displayed in the Supplementary Figure 5.


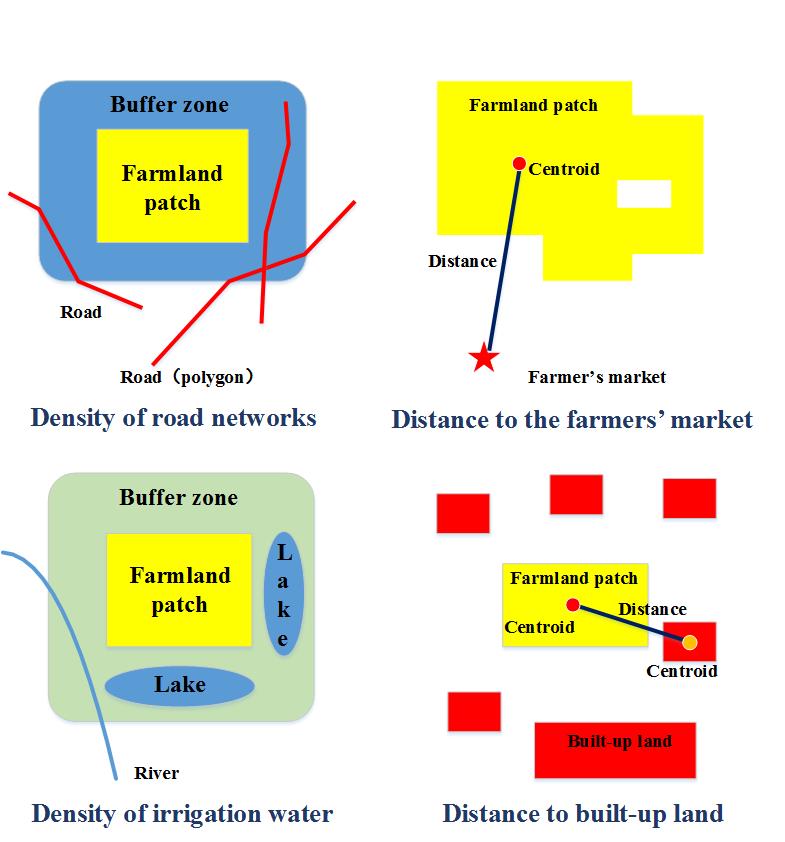


**Supplementary Figure 5.** Sketch map of the calculation of the four site assessment factors (the distance to built-up land, density of road networks, density of irrigation water, and distance to the farmers’ market).

(1) Density of road networks: calculated the road area of the unit area in the buffer zone of the farmland patch, higher values means the good traffic condition and get higher section scores;

(2) Distance to the farmers’ market: calculated the Euclidean distance between farmland patch and its closest farmer’s market, lower values indicate more convenient to conduct commodity trading and can get high section values;

(3) Density of irrigation water: calculated the water body area (mainly rivers and lakes) of the unit area in the buffer zone of the farmland patch, higher values means the good irrigation condition and get higher section scores;

(4) Distance to built-up land: calculated the Euclidean distance between centroid of the farmland patch and the closest built-up land patch, lower values indicate poorer agricultural environment and should get low section values.

### B3 Composite scores

After obtaining composite scores for each component, we synthesized the LE and SA components using different weight settings. Although the coefficient of LE and SA component should be generally equal, the influence of approximation algorithm in SA scores should be reduced. Finally, after some trial of ratio settings, a weight ratio of 2:3 was the optimal choice for following analysis, and the composite score (S_c_) of each farmland polygon was then calculated:

$Sc=0.4*LE+0.6*SA$ **(5)**

The composite scores for each farmland patch ranged from 0 to 10 and were displayed in Supplementary Figure 2-3.

## C. Spatial aggregation of PFs

The GIS spatial analysis for the demarcation of the PFPAs included a polygonal simplification algorithm, buffer analysis, and spatial aggregation.

### C1 Polygonal simplification algorithm

Because the remote sensing images are stored based on grid units, roughness and unsmoothness of the PF polygon boundary extracted from the satellite images is common and it can affect the accuracy of the spatial analysis ^[22]^. We used an improved Douglas–Peucker algorithm ^[23]^ to smooth and simplify the polygonal boundary. This robust method iteratively removes excessive nodes and minor banding without changing the area and shape. It produces correct results irrespective of the original polyline shape and tolerance parameter sizes ^[23]^. However, in this study, some additional manual corrections were performed to improve the accuracy. After this process, the PF patches, with smooth boundaries, could support the following spatial analysis (Supplementary Figure 6).


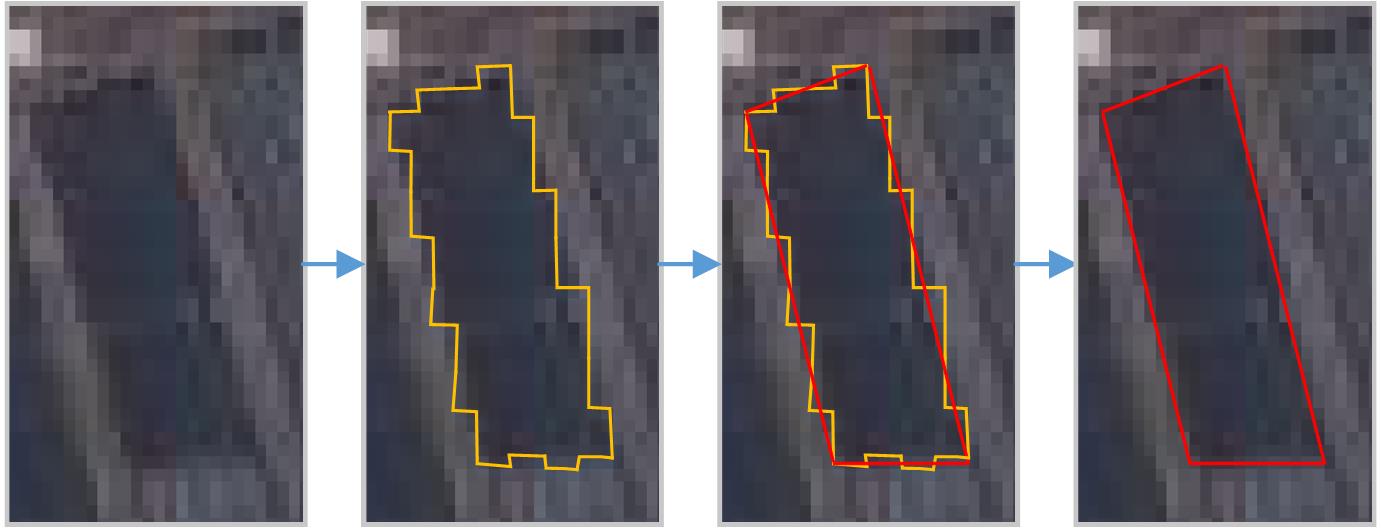


**Supplementary Figure 6.** Sketch map of the polygon simplification. The imageries were extracted from the satellite imageries (Figure 1) to amplify the imagery details (supported by SIGS (Worldview-2, 2013)). The figure was generated by N.X. using ArcMap 10.0 (<http://www.esrichina.com.cn/>. We have not used any map layer of the ESRI Company in this study).

### C2 Buffer analysis & Spatial aggregation

PF patches are not directly connected because of access roads for farm machinery, rural residences, and water conservancy facilities. As a basic GIS technique, buffer analysis establishes a strip domain of a certain distance around a geographic entity or space object to analyze the spatial neighborhood relationship ^[24]^ (Supplementary Figure 7). The basic idea for the construction of buffers is to identify the neighborhood of a certain spatial object ^[25]^. The buffer analysis could provide patches distance and adjacency conditions for the spatial aggregation.


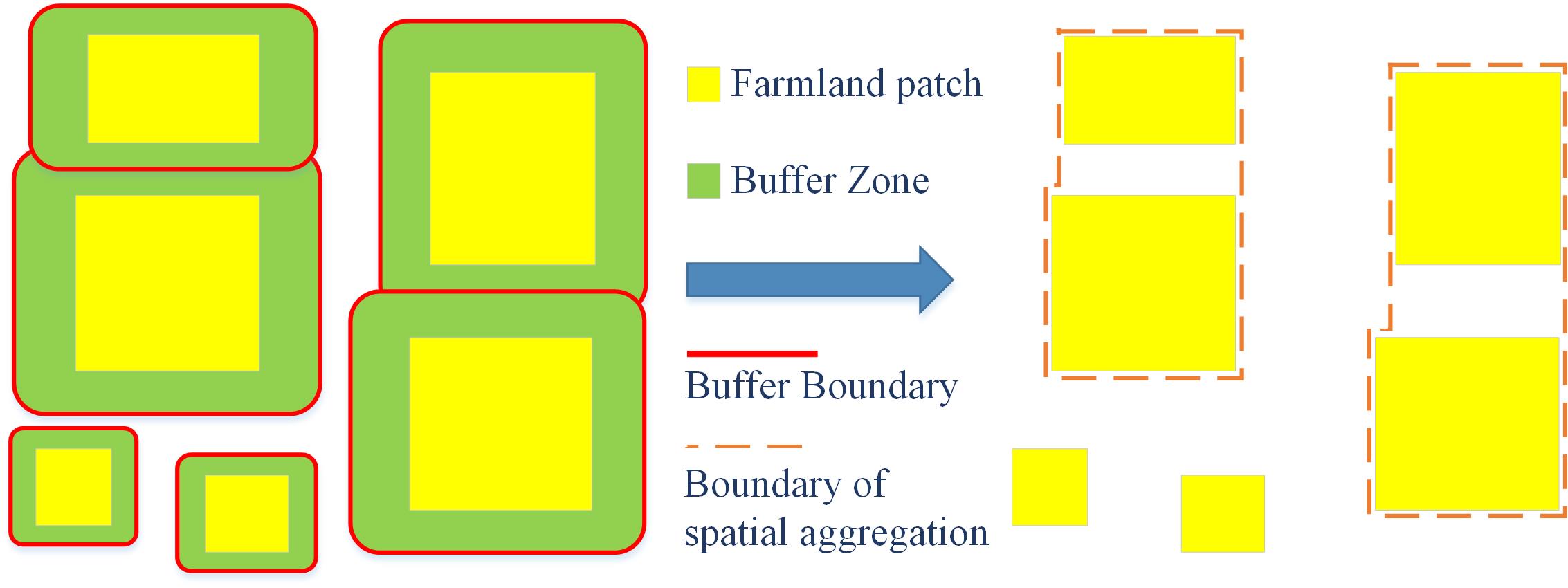


**Supplementary Figure 7.** Sketch map of the buffer analysis and spatial aggregation.

After identifying the proximity of PF patches by buffer analysis, we should outline the border of the PFPA. According to the Current Land Use Classification ^[26]^, PFPA could comprise some agricultural facilities, including furrows, canals, rural roads, and ditches. These facilities are indispensable parts of agricultural production and they should be protected together with PF (PF Regulations) ^[27]^. To overcome this problem, we used an iterative aggregation algorithm ^[28]^, which is a cartographic generalization method. Aggregation can eliminate the close tolerance between patches, and merge patches and their gaps into one single polygon ^[29]^ (Supplementary Figure 7). Only by defining the boundaries of patches at a certain distance (as confirmed by previous buffer analysis, *D*_b_) can aggregation happen. Connected and large PFPA patches should be given priority ^[27]^; therefore, we used a minimal area threshold (*A*_m_) value to eliminate discrete and relatively small PFPA patches (area < *A*_m_).

## D Dual threshold matrix analysis

### D1 Correlation analysis

5 of the 12 towns (CS, DT, LG, JH, and FC) are defined as suburban areas according to their spatial distribution, and validated by the matrices correlation (Supplementary Table 6). According to the statistical results of the proportion of PF demarcated in the PFPAs (pPF) under different threshold values (Supplementary Fig. 4, defined as dual-threshold matrices), it can be seen that obvious differences exist between the different towns. The image change regularity in CS, DT, LG, JH, and FC is similar. The correlations of the dual-threshold matrices between these five towns are relatively significant, and only strongly correlated with each other (Supplementary Table 6). Similarly, the remaining seven towns (MZ, GY, BH, LT, ZX, YX, and LX), which have similar change regularity in dual-threshold matrices, were defined as exurban areas.

### D2 Selection of the dual thresholds

The dual threshold matrix cannot directly determined the optimal value of distance threshold and area threshold, but can make us easy to understand the interaction characteristics of dual threshold. So we have to decide the optimal parameters based on the actual situation (distance cannot be too large to contain high-level roads or wide rivers), landscape connectivity of PFPAs (PFPAs cannot be too small), and the related policies (about 95% PFs had to be demarcated in the PFPA, pPF). That means we can use the pPF threshold (about 95%) to inversely decide the two thresholds. So the threshold settings in this study were not a pure scientific decision. It was a comprehensive decision involving subject judgement and objective circumstance.

For example, according to Figure 4a (composite result of dual threshold matrices for suburban areas), there are a lot of combinations of the two thresholds that can make 95% PFs in the PFPA (the green dotted curve area in supplementary figure 8). (1) The 1m distance threshold and 10 mu area threshold can demarcate 95% PFs in the PFPA. But that means only farmland patches within 1m can be aggregated into one piece (country road standard in China is 6m width). The rural road > 2m cannot be included in the PFPA. 10 mu means the demarcated PFPAs have a number of small regions, probably with one single small farmland patch that cannot be called as a PFPA. This setting would reduce the landscape connectivity of PFPAs. (2) The 15m distance threshold and 55 mu area threshold can also make 95% PFs in the PFPA. 15m means that the PFPA would contained some superior high roads (3.75m is the standard lane width in China, 15m means the double 4 lanes high-level roads). But PFPA could not contain these high-level roads for their polluting effects. Meanwhile, 55mu is too large for a PFPA (make it hard to manage), and there would be less farmland patches in a town.

That is to say, we can ensure that the distance threshold is 6-14m, the area threshold is 10-40mu, and the pPF is about 95%. With the combination of the physical implication of distance threshold, we decided distance threshold as about 9 meters in suburban area (the purple solid line in supplementary figure 8) and about 7 meters in exurban area according to the average country road width. Once the distance threshold and the pPF are determined, the area threshold can be determined within a certain extent. In other words, all the area thresholds in purple line (0-20mu, supplementary figure 8) can make the pPF > 95%. For the benefit of management and planning, we set the area threshold as 20mu to maximize PFPA areas (the blue intersection of purple line and green curve, supplementary figure 8). So the 9m and 20mu are the relatively optimal choice for the suburban area. It can include actual rural roads and remove some single small patches to improve the connectivity of PFPAs. Similarly, the specific thresholds were chosen for each towns which were based on some trials to make results more reasonable. We think the difference is enough obvious in a city.


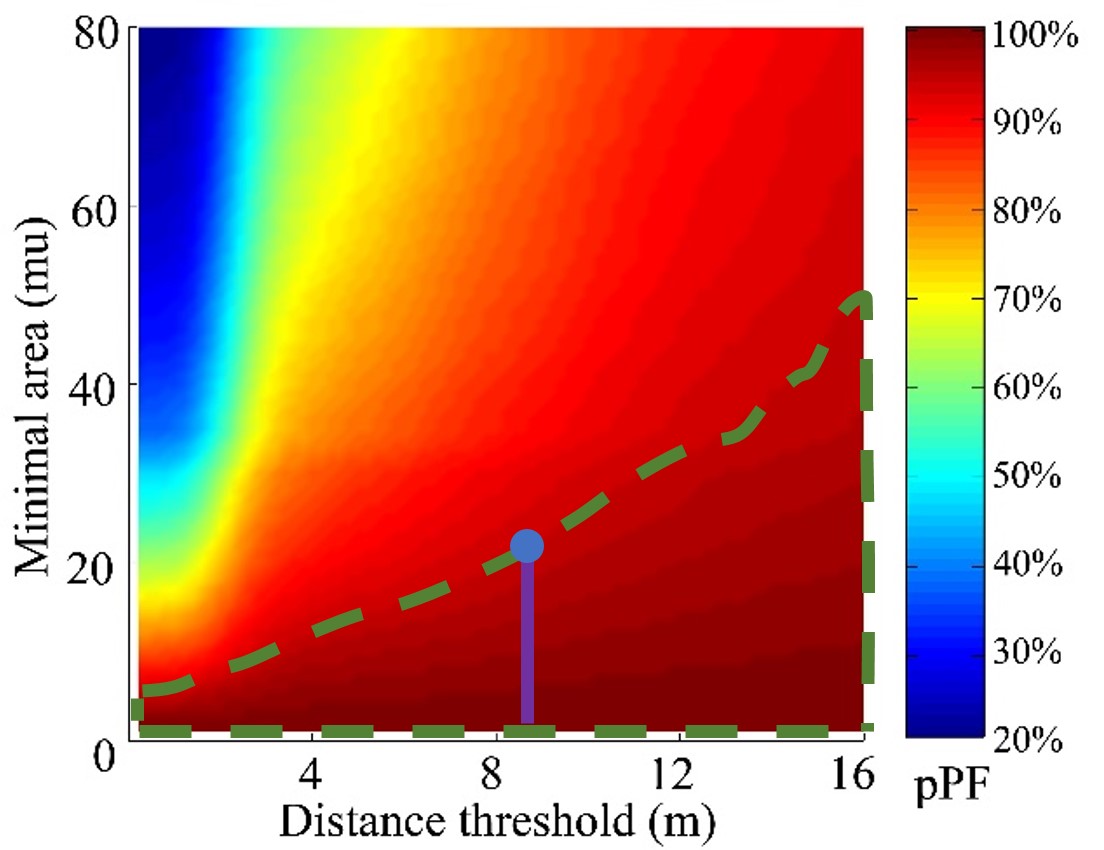


**Supplementary Figure 8.** The selection process of area threshold and distance threshold. The green dotted curve area means the combination of the two thresholds than can demarcate >95% PFs in the PFPAs. The purple line means the >95% PFs are demarcated in the PFPAs under 9 m distance threshold and 0-20 mu area thresholds. The blue point means the 95% PFs are demarcated in the PFPAs under distance threshold of 9 m and area threshold of 20 mu. The figure was generated by N.X. using Matlab R2014a (<http://cn.mathworks.com/> ).

## E. Landscape pattern indices

Although the spatial distribution of the demarcated PFPAs were expected to be less fragmented than the original PFs (by omitting small PFs and merging nearby ones), some non-farmlands were still included in the PFPAs during the demarcation, probably with tiny effect. So we think the calculation of the landscape pattern indices would be a better evidence to prove this apparent hypothesis. Since the selection of specific indices for different landscapes appears idiosyncratic, and no individual index can capture the full complexity of spatial patterns, highly contingent and multiple sets of indices are required ^[30]^. In this study, we selected the following 4 indices to reveal the PF landscape pattern comprehensively: These indices were all calculated by FRAGSTATS ^[31]^.

(1) The COHESION index (COHESION);

**(6)**

Where p_i_ is the perimeter of patch i, a_i_ is the area of patch i, n is the patch amount in the region. COHESION is sensitive to the aggregation of the focal class, and increases as the patches become more clumped or aggregated in distribution

(2) Effective Mesh Size

$\text{MESH}=\frac{\sum_{i=1}^{n} a_{i}^{2}}{A}$ **(7)**

Where a_i_ is the area of patch i, A is the total area of all patches. MESH has proven to monotonically decrease with increasing fragmentation, and is consistent throughout the fragmentation process.

(3) Landscape Shape Index (LSI)

**(8)**

Where e_i_ is the total edge length between patch i and background, A is the total landscape area. *LSI* =1 when the landscape consists of a single square patch, and increases without limit as landscape shape becomes more irregular.

(4) Perimeter-Area Fractal Dimension (PAFRAC)

$PAFRAC = \frac{\frac{2}{\left[n*\sum_{i=1}^{n} (lnp_{i}*lna_{i})\right]-\left[\left(\sum_{i=1}^{n} lnp_{i}\right)\left(\sum_{i=1}^{n} lna_{i}\right)\right]}}{\left(n*\sum_{\text{i}=1}^{n} lnp_{i}^{2}\right)-\left(\sum_{i=1}^{n} lnp_{i}\right)^{2}}$ **(9)**

Where p_i_ is the perimeter of patch i, a_i_ is the area of patch i, n is the number of patch. PAFRAC greater than 1 for a 2-dimensional landscape indicates a departure from a Euclidean geometry (an increase in patch shape complexity). PAFRAC approaches 1 for shapes with very simple perimeters such as squares, and approaches 2 for shapes with high convoluted, plane-filling perimeters.

**Reference**

1. Trimble. Trimble Documentation: eCognition Developer 9.0 Reference Book. Ch.30, 333-348 (Trimble Germany GmbH, 2011).
2. Ma, L., Cheng, L., Li, M. C., Liu, Y. X. & Ma, X. X. Training set size, scale, and features in Geographic Object-Based Image Analysis of very high resolution unmanned aerial vehicle imagery. *ISPRS-J. Photogramm. Remote Sens.* **102**, 14-27 (2015).
3. Benz, U. C., Hofmann, P., Willhauck, G., Lingenfelder, I. & Heynen, M. Multi-resolution, object-oriented fuzzy analysis of remote sensing data for GIS-ready information. *ISPRS-J. Photogramm. Remote Sens.* **58**, 239-258 (2004).
4. Blaschke, T. Object based image analysis for remote sensing. *ISPRS-J. Photogramm. Remote Sens.* **65**, 2-16 (2010).
5. Stehman, S. V. & Wickham, J. D. Pixels, blocks of pixels, and polygons: Choosing a spatial unit for thematic accuracy assessment. *Remote Sens. Environ.* **115**, 3044-3055 (2011).
6. Congalton, R.G. & Green K. Assessing the accuracy of remotely sensed data: principles and practices. CRC/Taylor and Francis Group, LLC, Boca Raton, London, New York (2009).
7. Radoux, J. & Bogaert, P. Accounting for the area of polygon sampling units for the prediction of primary accuracy assessment indices. *Remote Sens. Environ.* **142**, 9-19 (2014).
8. Pu, R. L. & Landry, S. A comparative analysis of high spatial resolution IKONOS and WorldView-2 imagery for mapping urban tree species. *Remote Sens. Environ.* **124**, 516-533 (2012).
9. Verikas, A., Gelzinis, A. & Bacauskiene, M. Mining data with random forests: A survey and results of new tests. *Pattern Recognit.* **44**, 330-349 (2011).
10. Laliberte, A. S. & Rango, A. Texture and Scale in Object-Based Analysis of Subdecimeter Resolution Unmanned Aerial Vehicle (UAV) Imagery. *IEEE Trans. Geosci. Remote* **47**, 761-770 (2009).
11. Immitzer, M., Atzberger, C. & Koukal, T. Tree Species Classification with Random Forest Using Very High Spatial Resolution 8-Band WorldView-2 Satellite Data. *Remote Sens.* **4**, 2661-2693 (2012).
12. Chan, J. C. W. & Paelinckx, D. Evaluation of Random Forest and Adaboost tree-based ensemble classification and spectral band selection for ecotope mapping using airborne hyperspectral imagery. *Remote Sens. Environ.* **112**, 2999-3011 (2008).
13. Puissant, A., Rougier, S. & Stumpf, A. Object-oriented mapping of urban trees using Random Forest classifiers. *Int. J. Appl. Earth Obs. Geoinf.* **26**:235-245 (2014).
14. Rodriguez-Galiano, V. F., B. Ghimire, J. Rogan, M. Chica-Olmo, and J. P. Rigol-Sanchez. An assessment of the effectiveness of a random forest classifier for land-cover classification. *ISPRS-J. Photogramm. Remote Sens.* **67**, 93-104 (2012).
15. Freire, S.et al. Introducing mapping standards in the quality assessment of buildings extracted from very high resolution satellite imagery. *ISPRS-J. Photogramm. Remote Sens.* **90**, 1-9 (2014).
16. Whiteside, T. G., Maier, S. W. & Boggs, G. S. Area-based and location-based validation of classified image objects. *Int. J. Appl. Earth Obs. Geoinf.* **28**, 117-130 (2014).
17. Allouche, O., Tsoar, A., & Kadmon, R. Assessing the accuracy of species distribution models: prevalence, kappa and the true skill statistic (TSS). *J. Appl. Ecol.* **43**, 1223-1232 (2006).
18. Ministry of Land and Resources of the People's Republic of China. The People's Republic of China national standards (GB/T 28407-2012): Regulations for gradation on agriculture land quality [S]. 2012
19. Ministry of Land and Resources of the People's Republic of China. The People's Republic of China national standards (GB/T 28405-2012): Regulations for classification on agriculture land [S]. 2012
20. Martellozzo, F. et al. Urbanization and the loss of prime farmland: a case study in the Calgary-Edmonton corridor of Alberta. *Reg. Environ. Change* **15**, 881-893 (2015).
21. Song, W., Pijanowski, B. C. & Tayyebi, A. Urban expansion and its consumption of high-quality farmland in Beijing, China. *Ecol. Indic.* **54**, 60-70 (2015).
22. Wade, T. G. et al. A comparison of vector and raster GIS methods for calculating landscape metrics used in environmental assessments. *Photogramm. Eng. Remote Sens.* **69**, 1399-1405 (2003).
23. Pallero, J. L. G. Robust line simplification on the surface of the sphere. *Comput. Geosci.* **83**, 146-152 (2015).
24. Emch, M. et al. Integration of Spatial and Social Network Analysis in Disease Transmission Studies. *Ann. Assoc. Am. Geogr.* **102**, 1004-1015 (2012).
25. Goodchild, M. F., & Hunter, G. J. A simple positional accuracy measure for linear features. *Int. J. Geogr. Inf. Sci.* **11**, 299-306 (1997).
26. Ministry of Land and Resources of the People's Republic of China. The People's Republic of China national standards (GB/T 21010-2007): Current Land Use Classification [S]. 2007
27. The State Council of the People’s Republic of China, 1998. Regulations on the Protection of Prime Farmland (Revised in 2011).
28. Haunert, J. H., & Wolff, A. Area aggregation in map generalisation by mixed-integer programming. *Int. J. Geogr. Inf. Sci.* **24**, 1871-1897 (2010).
29. Ai, T. H., Zhang, X., Zhou, Q., & Yang, M. A vector field model to handle the displacement of multiple conflicts in building generalization. *Int. J. Geogr. Inf. Sci.* **29**, 1310-1331 (2015).
30. Taylor, P., Fahrig, L. & With, K. A. (Eds). Landscape connectivity: back to the basics. In: Connectivity Conservation. Cambridge, UK: Cambridge University Press, 2006.
31. McGarigal, K., Cushman, S. A., Neel, M. C. & Ene, E. (Eds). FRAGSTATS: Spatial Pattern Analysis Program for Categorical Maps. Amherst: University of Massachusetts. www.umass.edu/landeco/research/fragstats/ fragstats.html, 2002.
